# The 3D Organization of the Yeast Genome Correlates with Co-Expression and Reflects Functional Relations between Genes

**DOI:** 10.1371/journal.pone.0054699

**Published:** 2013-01-31

**Authors:** Dirar Homouz, Andrzej S. Kudlicki

**Affiliations:** 1 Department of Biochemistry and Molecular Biology, University of Texas Medical Branch, Galveston, Texas, United States of America; 2 Institute for Translational Sciences, University of Texas Medical Branch, Galveston, Texas, United States of America; 3 Department of Applied Mathematics and Sciences, Khalifa University of Science, Technology and Research, Abu Dhabi, United Arab Emirates; 4 Sealy Center for Molecular Medicine, University of Texas Medical Branch, Galveston, Texas, United States of America; University of Minnesota, United States of America

## Abstract

The spatial organization of eukaryotic genomes is thought to play an important role in regulating gene expression. The recent advances in experimental methods including chromatin capture techniques, as well as the large amounts of accumulated gene expression data allow studying the relationship between spatial organization of the genome and co-expression of protein-coding genes. To analyse this genome-wide relationship at a single gene resolution, we combined the interchromosomal DNA contacts in the yeast genome measured by Duan et al. with a comprehensive collection of 1,496 gene expression datasets. We find significant enhancement of co-expression among genes with contact links. The co-expression is most prominent when two gene loci fall within 1,000 base pairs from the observed contact. We also demonstrate an enrichment of inter-chromosomal links between functionally related genes, which suggests that the non random nature of the genome organization serves to facilitate coordinated transcription in groups of genes.

## Introduction

The regulation of transcription in eukaryotes is a complex process that involves several levels of coordination. This regulation relies not only on *cis*-elements (such as transcription factor binding sites) but also on the genome organization at different scales, including the distribution of the nucleosomes, the folding of chromatin, as well as the chromosomal conformation and chromosomal territories [1], [2], [3], [4], [5], [6].

In the budding yeast, many aspects of nuclear organization have been observed in spite of its small genome. These include the placement [7] and folding [8] of chromosomes, clustering of telomeres [8], [9], [10], [11], [12], [13], the role of radial position within the nucleus [14], or the interactions between DNA and the nuclear envelope and association with the nuclear pores; for a comprehensive review see [15], [16], [17], [18].

Several recent studies [19], [20], [21], [22] suggest a direct involvement of these mechanisms in regulating transcription also in the budding yeast. Janga et al. [19] addressed the distribution of different transcription factors (TFs) in yeast among different chromosomes and showed that the targets of a TF tend to cluster on specific chromosomes.Nonetheless the existence of highly prevalent chromosomal territories in the budding yeast has been disputed [6], [23], [24].

Recent advances in experimental methods such as chromosome conformation capture (3C, 4C, 5C, 6C, and HiC) [3], [8], [25], [26], [27], [28] have made it possible to study the organization of DNA in the nuclear space with a high resolution. In genome-wide chromosome conformation capture experiments (4C, Hi-C) DNA fragments in close proximity are captured by fixation, digestion, and intra-molecular ligation. These DNA fragments are then sequenced to determine the two loci involved in each contact. The number of sequenced fragments for each contact is reported as the “count frequency”, which is interpreted as a measure of spatial proximity between genomic loci, which in turn allows modelling the 3D structure of the entire genome [22], [28]. In the fission yeast *Schizosaccharomyces pombe*, evidence points to enrichment of several Gene Ontology (GO) groups among genes with contacts between their loci, as well as to co-expression of such genes during the G2 phase of the cell division cycle [29]. In the budding yeast *S. cerevisiae*, Duan et al. [28] have mapped the DNA contacts (links) across all chromosomes using the 4C method. These contact maps were used in building a 3D model of the yeast genome, which shows several trends that point to the existence of chromosomal territories in the budding yeast.

In this work, we analyzed the experimental data of Duan et al. [28] in order to investigate the connection between these contacts in the budding yeast and the co-expression of protein-coding genes across a large collection of expression datasets representing a broad range of conditions. Our results show that the measured expression levels of genes localized near inter-chromosomal links are significantly correlated. This correlation increases for pairs of genes with stronger links, as inferred from the higher frequency of counts in the 4C experiment. Our analysis demonstrates an enrichment of inter-chromosomal links connecting loci of genes with the same GO term. This in turn suggests that the spatial organization of the yeast genome is non-random and facilitates coordinated expression of functionally related genes.

## Results

The spatial contacts between different parts of the yeast genome identified by 4C captured links are both intra- and inter-chromosomal. The co-expression of genes located on the same chromosome may be affected by *cis-* effects due to sequence proximity. Here, we restrict the analysis to links between loci on different chromosomes, which allows focusing on the interchromosomal interactions, and also eliminating the influence of any cis- effects.

### Inter-chromosomal Contacts and Co-expression of Genes

**Figure 1:**
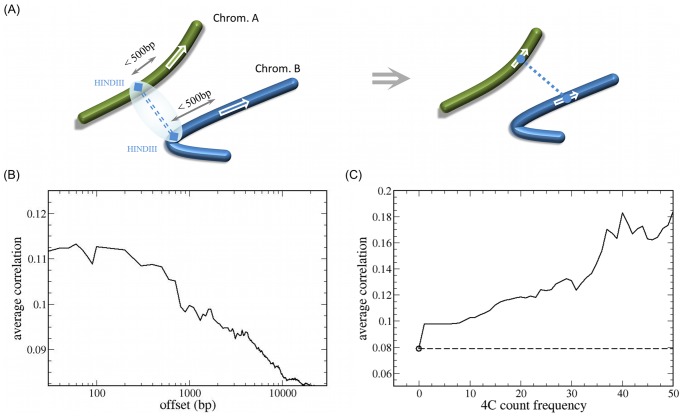
Interactions between genomic loci and correlation of expression profiles. (A) The interaction between gene loci is inferred from the existence of an experimental 4C link between two HINDIII sites on two different chromosomes within genomic separation of less than 500 base pairs from the genes. (B) The average correlation of genes as a function of the distance (offset) from an inter-chromosomal contact. The contacts are based on the HINDIII library of the experimental data. The correlations between the corresponding genes are calculated based on 1496 Affymetrix Yeast S98 microarray samples obtained from the GEO database. (C) The average correlation between linked genes depends on the experimental count frequency threshold (number of detected fragments) of the corresponding links. Frequency of zero corresponds to all possible pairs of genes (linked and unlinked) and the represents the genome wide average for all inter-chromosomal pairs of genes. The genome wide average is highlighted here by the circle and the horizontal dashed line for improving the visual comparison. The number of contacts is based on the HINDIII library.

For all pairs of genes potentially affected by the inter-chromosomal contacts, we computed the average Pearson correlation coefficient of expression level (transcript concentration) over a comprehensive set of microarray measurements in 1496 experimental conditions, see Methods. The average correlation coefficient for interacting loci was then compared to the whole-genome average. We defined the pairs of genes with contacts as those located within a specified offset of both ends of a 4C captured link (see Figure 1.A). The dependence on the size of this offset is shown in Figure 1.B, the correlation is high for small offsets (<500 bp) and decreases as the offset increases until it reaches a plateau around 10 kbp. The curve has a shoulder near 500 bp which we adopt as the threshold for our subsequent calculations. We find that the correlation between linked genes (0.0977) is significantly higher than genome wide average (0.0789, p-value <10^−317^, KS test). Computing the average correlation for a window with the same offset, but centred on different positions with respect to the 5′ end of the coding sequence of a gene shows a slight asymmetry towards the region upstream of the beginning of the gene, however this result is not definitive because of the high level of shot noise in the signal (Supplementary Figure S.1).

We also calculated the average co-expression for genes connected by links as a function of the threshold count frequency in the 4C experiment. We found that the correlation increases monotonically with the frequency of the experimental fragment count for the contact (related to the probability of contact) as it can be seen from Figure 1.C. This result demonstrates the significant association between gene co-expression and proximity in the nuclear space.

The dependence of correlation in gene expression on inter-chromosomal interactions also manifests itself through the average correlation coefficients between transcripts on different pairs of chromosomes. We find them to be significantly correlated (cc = 0.415; p-value = 5×10^−7^, one-tailed *t*-test) with the average number of links between chromosomes per kbp of chromosome length for the same pairs (see Supplementary Figure S.2).

### Average Expression of Linked Genes

The relation between the DNA links and the global expression profiles can be analysed to find any dependence between average expression of a gene and the enrichment of its neighbourhood in genomic contacts. To this end, we bin all genes into groups according to their average expression rank and compute the average enrichment in links for the genes in each bin. We find that genes with both very low and very high expression are depleted in links, while genes with more typical expression levels are enriched in links. The relation is shown in Supplementary Figure S.3.

### Inter-Chromosomal Contacts and Go Terms

The results presented above establish the significant relationship between the genome contacts and the co-expression of genes. One can postulate three different models of the causal nature of this relationship:

- the three-dimensional structure constitutes a mode of gene regulation which is selected in evolution and complements other regulatory mechanisms, e.g. regulation of expression by recognizing transcription factor binding sites.
- it is only a secondary effect and we observe the links *because* the genes are coexpressed and therefore brought together to the “transcription factories” within the yeast nucleus.
- The three-dimensional structure of the genome does influence gene expression, however this regulation does not serve a genome-wide biological function and the correlations in expression are only a side effect of an arbitrary conformation of the chromatin.

**Figure 2:**
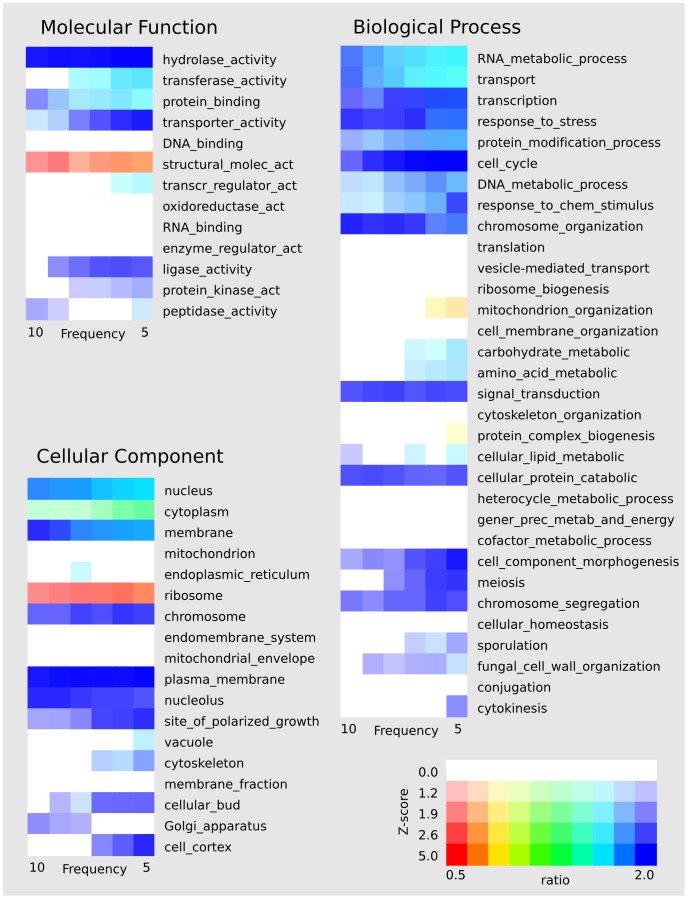
The distribution of inter-chromosomal contacts within groups of genes by GO-slim terms. The distribution is characterized by the ratio of the observed number of linked genes for each GO term to that of the number predicted from Monte Carlo simulation. The ratio is shown here by the hue of the color, where blue and purple correspond to high ratios (or enriched terms) and orange and red to low ratios (depleted terms). The significance of the ratio is represented here by the saturation of the colour as shown in the legend. The GO terms are divided into the three main domains and sorted according to their number of genes. The ratios are provided for all terms at different threshold count frequencies in the experimental link data with the two libraries combined.

While our data cannot resolve between the first two possibilities (the causation between regulation and conformation), we were able to rule out the third one and show that the links actually do correlate with the biological functions of the affected loci. To this end, we analysed the distribution of the chromosomal contacts within groups of genes with similar annotations. Specifically, we compared the number of contacts within the top level gene ontology (GO-slim) [30] terms with that of randomly selected groups with the same number of genes (see Supplementary Table S.1). Figure 2 shows the enrichment of inter-chromosomal links among different GO terms at different threshold count frequencies, computed using data from both –HINDIII and EcoRI libraries (the results for the HINDIII library only show a similar trend and are shown in Supplementary Figure S.4). The GO terms are divided into the three main domains (molecular function, biological process, and cellular component) and ordered according to the number of genes in each domain. Most of the terms in each of the three domains are significantly enriched with inter-chromosomal contacts. The enrichment ratio of the different terms also tends to be more pronounced at higher threshold frequencies defining strengths of contacts (see Supplementary Figure S.5). However, for most GO terms the number of contacts with higher count frequencies is low and the significance of this trend is reduced due to the Poissonian noise.

Among the terms with the highest ratios of enrichment are processes known for being regulated by transcription factors, such as cell cycle [31], [32] and stress response [33]. In the cell cycle genes, the number of observed contacts (at 4C frequency >5) is 1.73 times the expected number of contacts for a random group of the same number of genes (521) and this enrichment is statistically significant (p-value = 8×10^−9^). In the “response to stress” genes, the enrichment of contacts is also highly significant (number of genes = 561, ratio = 1.43 and p-value = 2×10^−3^).

Only very few terms show significant depletion of contacts, and they include non-specific terms, as the group of all genes annotated as “dubious” which is significantly depleted of contacts (number of genes = 787, ratio = 0.54 and p-value = 2×10^−7^). The only truly functional term with significant depletion are the ribosomal genes (i.e. transcripts coding ribosomal proteins), however this behaviour may be explained by the high average expression of these genes, as well as the fact that the corresponding GO term contains both mitochondrial ribosomal and cytosolic ribosomal genes.

**Figure 3:**
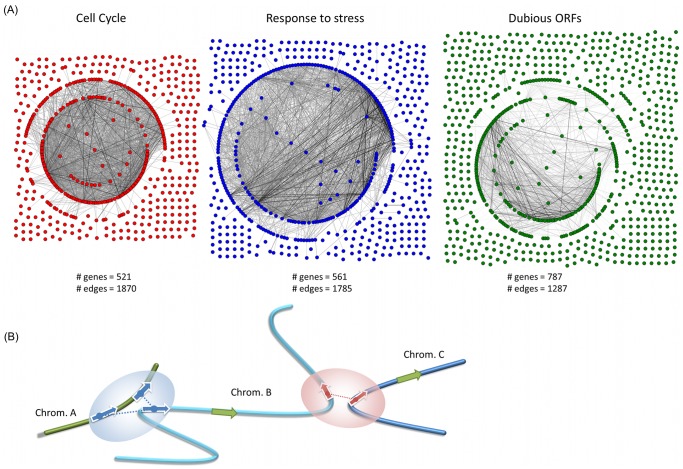
The 4C contact networks in yeast. A) The topology of 4C contact networks for three different groups of genes (“cell cycle” in red, “response to stress” in blue, and dubious ORFs in green). The contacts shown have a 4C frequency over than 5 and the shade of grey corresponds to the frequency of each contact. (B) A schematic diagram of the relation between inter-chromosomal contacts and regulation of genes from different functional groups. Genes with the same GO term (red or blue) tend to co-localize near the contacts while non-annotated and inactive genes (green) tend to avoid these links.

The distribution of links between genes in a specific functional group can be also represented as a “contact network”, or an undirected graph of links between the genes in the group. Figure 3.A shows three such graphs for “cell cycle” genes, “response to stress”, and for dubious ORFs. The topologies of these three contact networks highlight the difference between groups enriched with inter-chromosomal links and the depleted ones. For the cell cycle and stress response networks, most of the genes cluster in one big connected subgraph with a large number of links. The ratio of the number of edges per node is 3.6 in the cell cycle contact network and 3.2 in the stress response contact network. On the other hand, in the graph for the dubious genes, most of the nodes are not connected and the major subgraph is sparse in comparison to the other two graphs (1.6 edges per node) despite containing a larger number of genes. The graphs for gene-gene contact networks of all GO-slim terms are provided in the Supplement.

## Discussion

The co-expression of genes that are in spatial proximity provides evidence for a role of the genome conformation in regulation of gene expression, although it could not be determined if the role is a primary or a secondary one. We have demonstrated that functionally related genes cross-link together more often in the nuclear space, as do genes with similar expression profiles (Figure 3.B). This effect is the strongest for genes whose average expression levels are neither very high nor very low.

The dependence between gene regulation and the spatial organization of the genome has been very well established in several higher eukaryotes [2], [3]. In these organisms the chromosomes occupy specific regions of the nuclear space called “chromosomal territories”. The flexibility of chromosomal arms and the existence of many interactions between different chromosomes have suggested that budding yeast lacks chromosomal territories [23]. On the other hand, the so called “gene territories” [24] and the observed regulatory aspects of the genome spatial organization [19] support the hypothesis that chromosomal territories do exist in yeast. The 3D model of the yeast genome published by Duan et al. [28] suggests confinement of each chromosome to a specific region of the nucleus, although the authors do not address the question of territories directly, but instead highlight the flexibility of the chromosomal arms. In analysing the connections between the expression, spatial position and function of a gene, we have provided evidence that the interchromosomal DNA interactions are non random at the scale of the individual genes. Our results suggest that yeast chromosomes will assume specific conformations to facilitate gene co-expression and even if the conformation may be dynamically changing, the loci of coexpressed genes will tend to spend a significant fraction of time in proximity within the nucleus.

Both telomeric and centromeric regions of all yeast chromosomes group together in the nuclear space [22], [34], [35], [36]. To estimate the contribution of transcripts localized in these regions to the observed co-expression of interacting loci, we have computed the average correlations between expression profiles of genes grouped into bins according to their relative distance between the centromere and telomere. The set contains 2.38*10^6^ pairs of genes (approximately 10% of the total 2.3*10^7^ pairs in the genome) divided into 10 bins. The findings, presented in Supplementary Figure S.6, demonstrate that co-expression in these colocalized regions is too weak to explain the global co-expression of interacting loci. This constitutes additional evidence that the conformation on the scale of the individual genes is the main factor responsible for the observed co-expression.

Evidence of a genome duplication in *Saccaromyces* has been demonstrated [37], however only approximately 500 homolog pairs created in this event remain in *S. cerevisiae*, and these homologs diverge in expression patterns and regulatory regions [38], so they are not expected to significantly contribute to the average co-expression of interacting or non-interacting genes. Indeed, the contribution of these homologs to the genome-wide average correlation is negligible: removing pairs of homologous genes from the analysis changes the average correlation by 4×10^−6^. We have also removed one homologous gene from every pair and repeated the analysis disregarding the correlations between the removed genes and the rest of the transcriptome. The resulting change in the average correlation for either interacting or non-interacting pairs was less than 0.001, the exact number depending on which homologs were excluded from the analysis. This demonstrates that the duplicated genes do not contribute significantly to the global dependence between genome structure and gene co-expression.

Eukaryotic genomes are also known to be compartmentalized into spaces for active and inactive genes [2], [3], [39]. The difference between the positioning of active and inactive genes is reflected in yeast by the enrichment of 3D contacts for highly regulated genes and the depletion for dubious ORFs. We show that the group of all dubious ORFs (787) in the yeast genome is significantly depleted of contacts (p-value = 2×10^−7^). This fact may indicate that those dubious, and often inactive, genes are displaced away from the links that exist between active genes (Figure 3.B).

### Conclusions

We have confirmed that the 3D conformation of the yeast genome is non-random at the scale of individual genes. Based on the experimentally measured 4C contacts, we have presented a global picture of genome organization which is reflected by the gene-gene contact (interaction) networks within various functional groups. The understanding of the spatial aspect of genome regulation will be further enhanced with the development of experimental and computational methods and the availability of more high resolution genome-wide data.

The existence of a regulatory function of genome organization in yeast suggests that yeast may be a valid model organism for studying the mechanisms regulating the spatial structure of the genome.

## Methods

### Analysing 4C Contact Data

The experimental data for genome wide contacts in yeast are obtained from the work of Duan et al. The cross-linked DNA was digested using two different restriction enzymes (HINDIII and EcoRI) and thus the data is divided into two libraries. The number of inter-chromosomal contacts with frequency of 5 or higher as reported using the HINDIII library is 240629 and in the EcoRI library it is 72860. The HINDIII library has been used for all calculations in this work. The same calculations were also performed on the EcoRI library and the results which are qualitatively and quantitatively similar, although at a lower resolution and a lower confidence level, are provided in the Supplement (Figure S.7). The two libraries were combined in order to improve the statistics of the GO term contact distributions. We processed the inter-chromosomal contacts by mapping them to the corresponding genes in the SGD features data base [40]. A gene is assigned to a locus if it lies within an offset from the genomic position of that locus. In this work, we used an offset of 500 base pairs (see Figure 1.A).

Several sources of experimental bias may affect a 4C experiment [41], [42], [43], however the 4C data of Duan et al. [28] have been controlled for such effects using a number of methods, including assessing random inter-molecular ligations from five control libraries, controlling restriction site-based biases, testing reproducibility between independent sets of experimental libraries that differed in DNA concentration at the 3C step, verifying consistency between the HindIII and EcoRI libraries, and comparing the results with conventional 3C experiments. Moreover, while experimental biases may be significant when the interactions of a single locus are analysed, in the present study we focus on of linkage properties of large groups of genomic loci and any such effects are expected to average out. To confirm this, we repeated the co-expression analysis using DNA interaction data normalized with three different methods [43]: Sequential Component Normalization (SCN) [43], linear [44], and Euclidean [43]. In these methods, the 4C contact map is represented as a two dimensional matrix. In linear and Euclidean normalizations, each matrix element is divided by the product of the corresponding row and column sums or Euclidean norms, respectively. Finally, the SCN method works by symmetrizing the contact matrix through an iterative procedure which normalizes rows and columns to one.

We computed the average correlation of expression profiles in 200,241 gene pairs affected by the strongest interchromosomal interactions in each of these three normalized datasets and compared them to the interactions with frequency 5 or higher in the original data. The average correlations are respectively 0.099, 0.098 and 0.099 and remain very close to the number 0.098 obtained without any additional normalization. Similarly, for 51,849 strongest normalized interchromosomal links, the average correlations are 0.099, 0.096 and 0.099, also not significantly different from the figure of 0.101 obtained for the original data, (for links with frequency 8 or higher). All of the averages, for every normalization method are significantly higher that the genome average of 0.079, which demonstrates that our results do not depend on the normalization method used in data pre-processing.

### Yeast Microarray Gene Expression Data and Co-Expression Analysis

The yeast gene expression data were obtained from the GEO website [45]. We have used data collected with the Affymetrix yeast platform S98, covering a wide range of experimental conditions. The total number of samples used in our analysis was 1496, the complete list of sample accession numbers is provided in Supplementary Table S.2. All the data samples are normalized by converting to the linear scale and then dividing by the sample mean.

In order to quantify the co-expression of two genes, we calculated the Pearson correlation coefficient between the corresponding probes across all 1496 samples. This calculation has been performed for all pairs of genes to calculate the genome wide correlation average as well as the average correlation for linked genes at different contact strength thresholds.

To demonstrate the statistical significance of the co-expression of interacting genes, we have performed two simulations to estimate the effect of variance within the genome and within the population of interacting loci. First, we generated an ensemble of 30,000 control experiments, with randomly selected DNA interactions in the same number as in the actual experimental data, and repeated the correlation analysis in every one of them. As a result, we find that the average correlations for these simulated linkage sample is 0.0790 with a standard deviation of 0.0004. Second, we did a bootstrap analysis of data consistency, by using only 50% of the measured interactions, and repeating the analysis 1,000 times. The distribution of thus obtained average correlations is very close to a Gaussian with an average of 0.0983 and standard deviation equal 0.0005. The results of both simulations demonstrate that the observed co-expression of genes associated with interacting loci cannot be a result of a statistical fluctuation, and are biologically significant; the findings are summarized in Supplementary Figure S.8.

### Go Terms Enrichment Analysis

The enrichment of GO-slim terms is determined by counting the number of contacts between all the genes belonging to each term and comparing it to the number expected for gene interactions that do not depend on functional category. The expected numbers of links are obtained for all GO-slim terms from Monte Carlo simulations. For each term we generate 1,000 groups of genes randomly selected from the genome. The number of genes in each random group is equal to the number of genes annotated by the term of interest. The 4C links are counted between all pairs of genes in this group as for the original data, and the average and distribution over the 1000 simulations define the expected statistical properties of links for each GO category.

## Supporting Information

Figure S1 **The average correlation for a window with a size of 4000 bp centred on different positions with respect to the 5′ end of the coding sequence.**
(PDF)

Figure S2 **The average coexpression between a pair of chromosomes (calculated based on the correlations between the measured expression levels of all pairs of genes in the two chromosomes) versus the number of measured experimental contacts (intra- and inter-chromosomal in the HINDIII library) between the two chromosomes per 1000 base pairs (kbp).** The red line shows the linear regression with a correlation coefficient of 0.415 (p-value = 5×10^−7^).
(PDF)

Figure S3 **The distribution of inter-chromosomal contacts among genes as a function of their average expression rank.** The average expression rank is calculated for groups of 500 genes each. The contact enrichment for each group is the ratio of the number of observed contacts to that of the predicted number.
(PDF)

Figure S4 **The distribution of inter-chromosomal contacts (HINDIII library only) within groups of genes with different GO-slim terms.** The distribution is characterized by the ratio of the observed number of linked genes for each GO term to that of the predicted number. The ratio is shown here by the hue of the colour, where blue corresponds to high ratios (or enriched terms) and red to low ratios (depleted terms). The significance of the ratio is represented here by the saturation of the colour. The GO terms are divided into the three main domains and sorted according to their number of genes. The ratios are provided for all terms at different threshold count frequencies in the experimental link data.
(PDF)

Figure S5 **The average ratio (observed/expected links) for the three domains of GO (Molecular Function in black, Biological Process in red, and Cellular Component in green) as a function of the frequency of the 4C linkage data.** The figure (a) shows the average for enriched terms and (b) for depleted terms.
(PDF)

Figure S6 **The coexpression of interacting genes cannot be explained by telomere or centromere clustering. Blue solid line: The average correlation of expression profiles for all interchromosomal gene pairs in the genome.** Green solid line: The average correlation of expression profiles for pairs of genes associated with DNA interactions measured by 4C. Red points: The average correlation of expression profiles within groups of genes with similar relative position between the centromere and telomere.
(PDF)

Figure S7 **The average correlation between linked genes as a function of the experimental count frequency of the corresponding contacts based on the EcoRI library.** Frequency of zero corresponds to all possible pairs of genes (linked and unlinked) and represents the genome wide average for all possible inter-chromosomal pairs of genes. The genome wide average is highlighted here by the circle at the horizontal dashed line for improving the visual comparison.
(PDF)

Figure S8 **The significance of coexpression of genes associated with interacting loci.** Black: The histogram of 30,000 average correlation coefficients within groups of randomly chosen genes, each generated by choosing 240629 pairs of genes from the entire genome. (green line shows the genome average). Red: The histogram of 1000 average correlation coefficients between linked genes, generated by bootstrapping (choosing a random subset of 120300 interactions between linked genes). Blue line shows the average of all interacting genes.
(PDF)

Table S1 **A listing of the number observed 4C contacts for all GO-slim terms versus the expected number.** The numbers are calculated at different threshold count frequencies. Monte Carlo simulations are used to generate 1000 random samples for each term. The expected number of contacts is determined from the average number of contacts in the 1000 samples and the standard deviation gives the Z-score.
(PDF)

Table S2 **This table lists the GEO accession numbers for 1496 gene expression microarray samples used in this work.**
(PDF)

File S1 **Contact networks for GO-slim terms.** The figures show the contact networks (frequency >5) for each of the Go-slim terms. The number of links per gene is shown below each figure.
(PDF)
