# Supplementary figures and images for: The 3D Organization of the Yeast Genome Correlates with Co-Expression and Reflects Functional Relations between Genes
Source: PLoS One. 2013 Jan 31;8(1):e54699. doi: 10.1371/journal.pone.0054699 (PMC3561378; doi:10.1371/journal.pone.0054699)

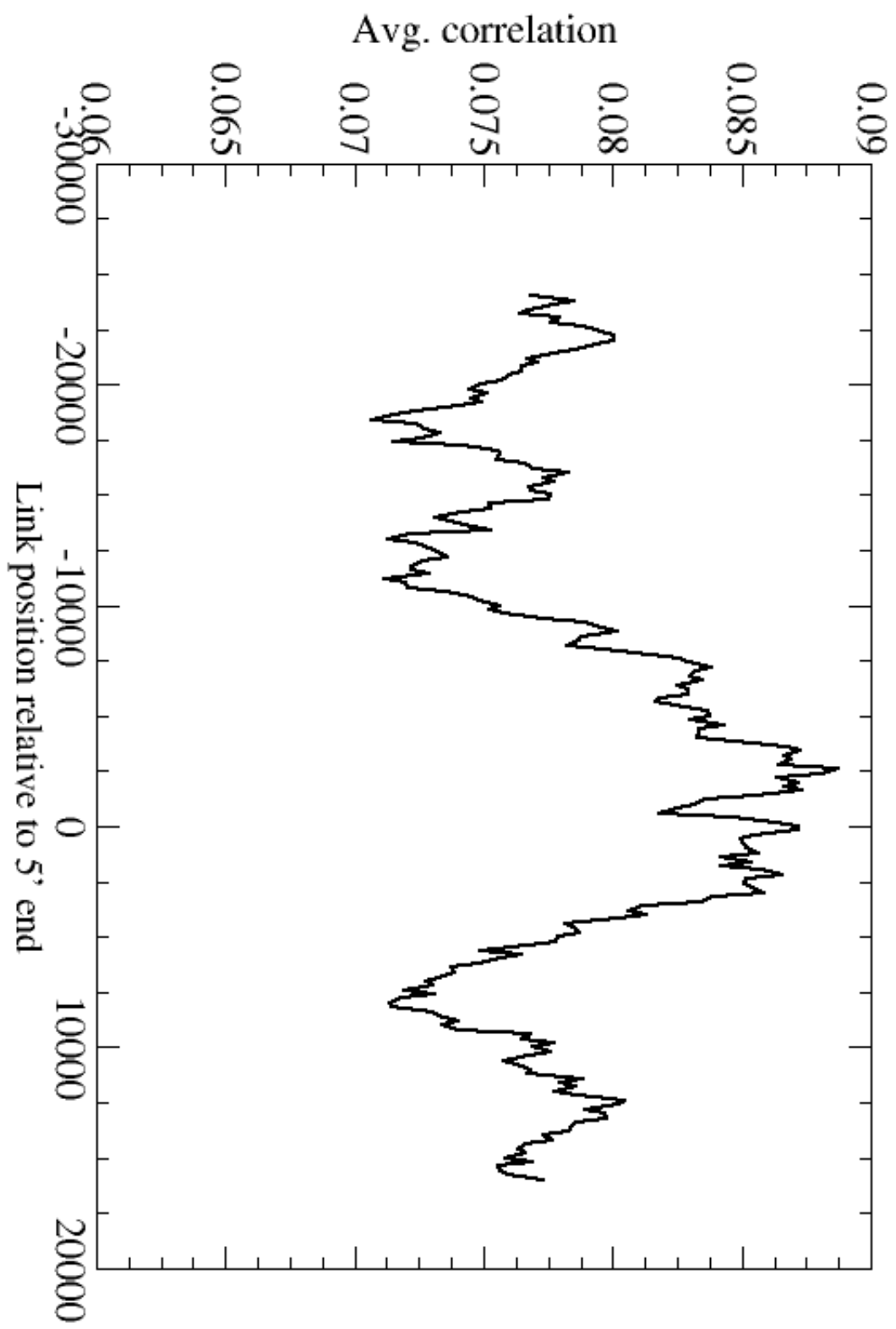

Supplement: Figure S1 — The average correlation for a window with a size of 4000 bp centred on different positions with respect to the 5′ end of the coding sequence. (PDF) [file pone.0054699.s001.pdf]

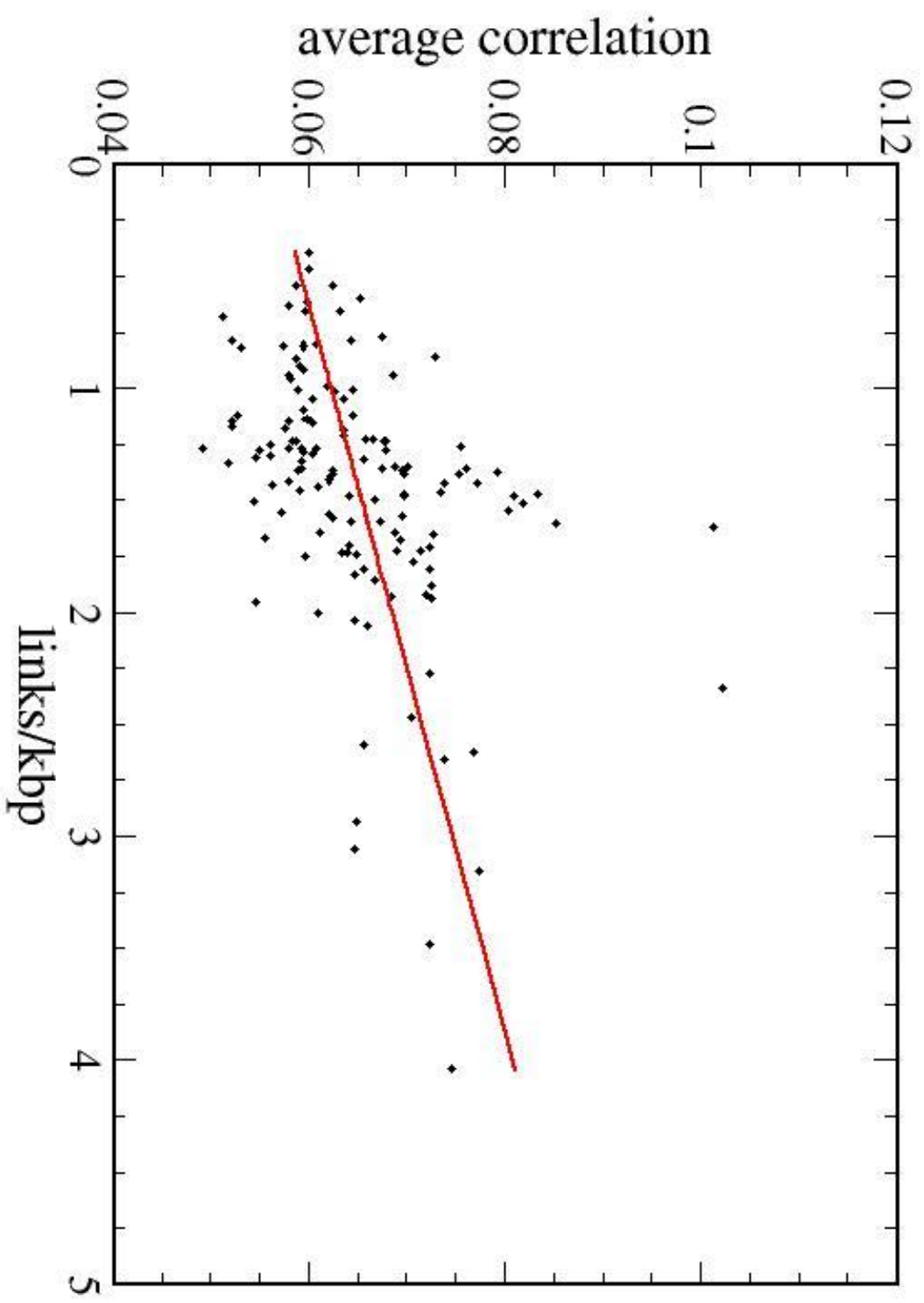

Supplement: Figure S2 — The average coexpression between a pair of chromosomes (calculated based on the correlations between the measured expression levels of all pairs of genes in the two chromosomes) versus the number of measured experimental contacts (intra- and inter-chromosomal in the HINDIII library) between the two chromosomes per 1000 base pairs (kbp). The red line shows the linear regression with a correlation coefficient of 0.415 (p-value = 5×10^−7^). (PDF) [file pone.0054699.s002.pdf]

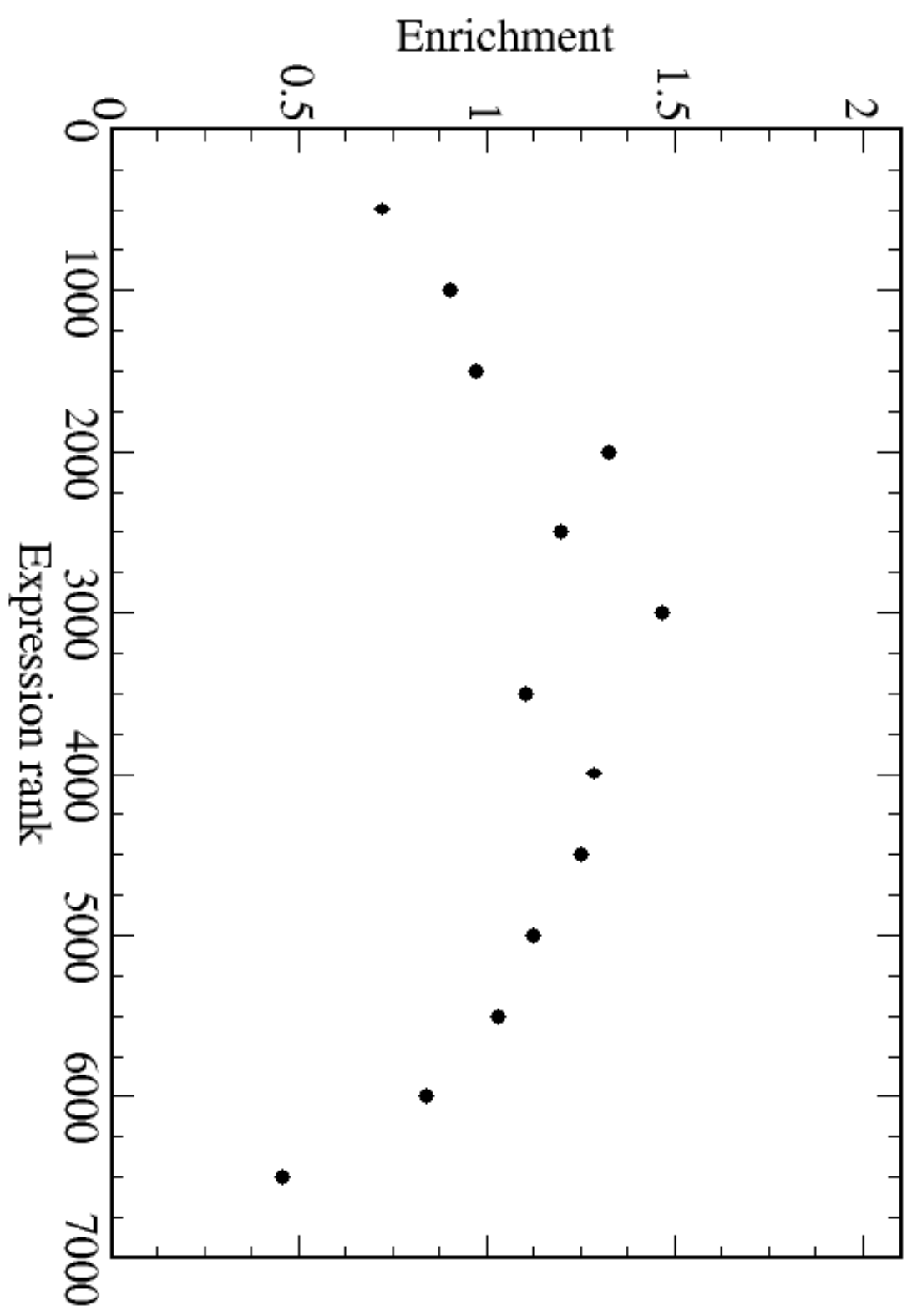

Supplement: Figure S3 — The distribution of inter-chromosomal contacts among genes as a function of their average expression rank. The average expression rank is calculated for groups of 500 genes each. The contact enrichment for each group is the ratio of the number of observed contacts to that of the predicted number. (PDF) [file pone.0054699.s003.pdf]

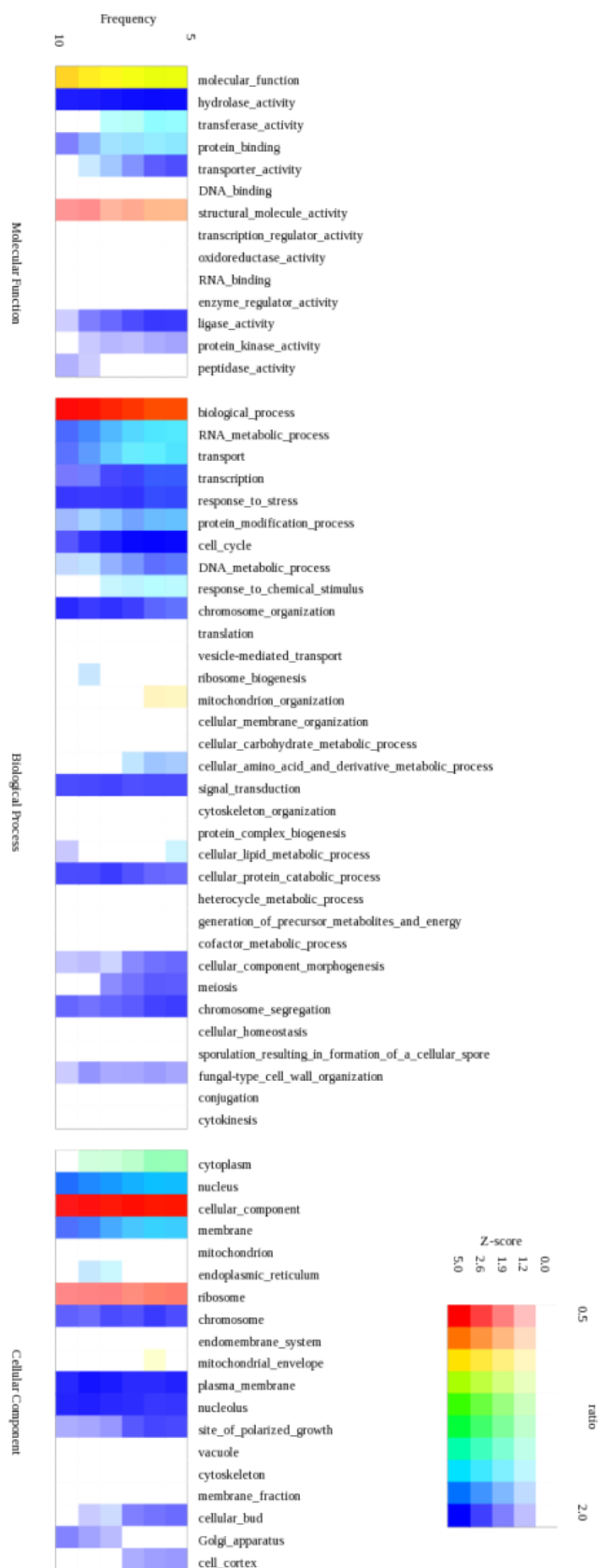

Supplement: Figure S4 — The distribution of inter-chromosomal contacts (HINDIII library only) within groups of genes with different GO-slim terms. The distribution is characterized by the ratio of the observed number of linked genes for each GO term to that of the predicted number. The ratio is shown here by the hue of the colour, where blue corresponds to high ratios (or enriched terms) and red to low ratios (depleted terms). The significance of the ratio is represented here by the saturation of the colour. The GO terms are divided into the three main domains and sorted according to their number of genes. The ratios are provided for all terms at different threshold count frequencies in the experimental link data. (PDF) [file pone.0054699.s004.pdf]

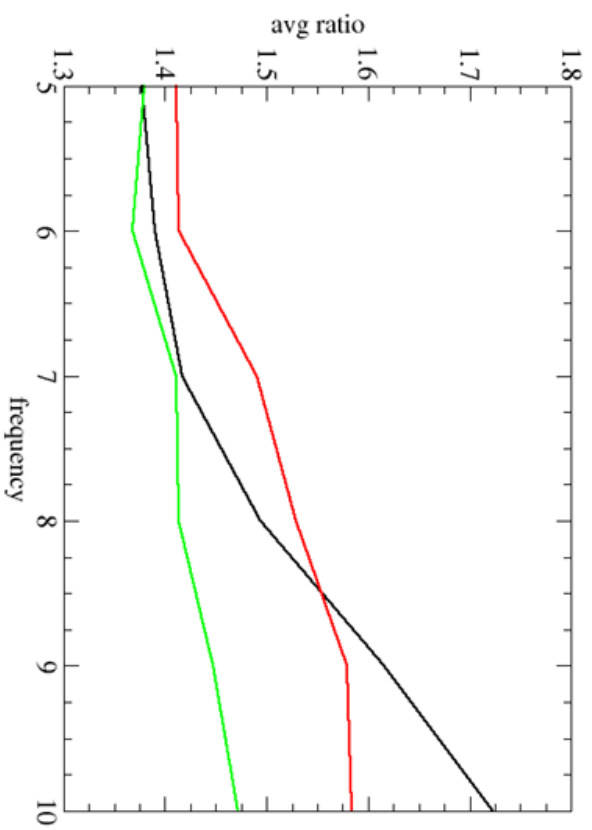

(a)

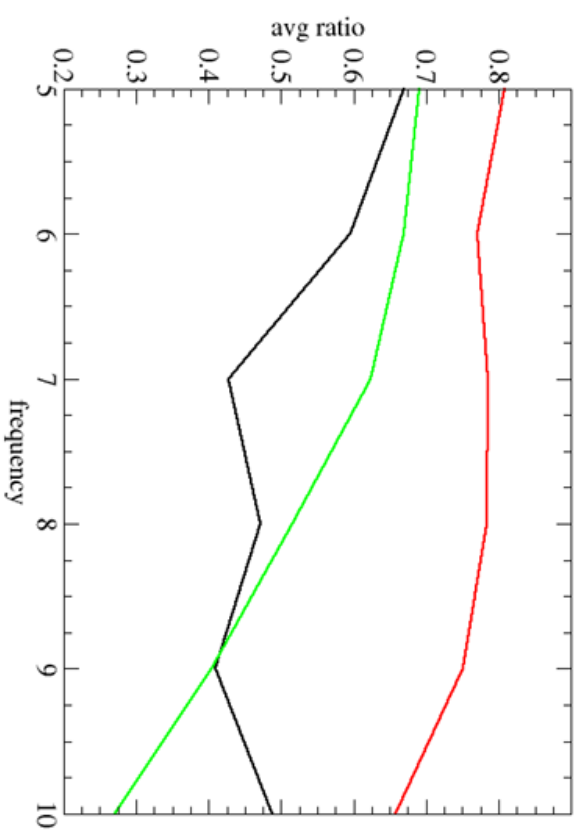

(b)

Supplement: Figure S5 — The average ratio (observed/expected links) for the three domains of GO (Molecular Function in black, Biological Process in red, and Cellular Component in green) as a function of the frequency of the 4C linkage data. The figure (a) shows the average for enriched terms and (b) for depleted terms. (PDF) [file pone.0054699.s005.pdf]

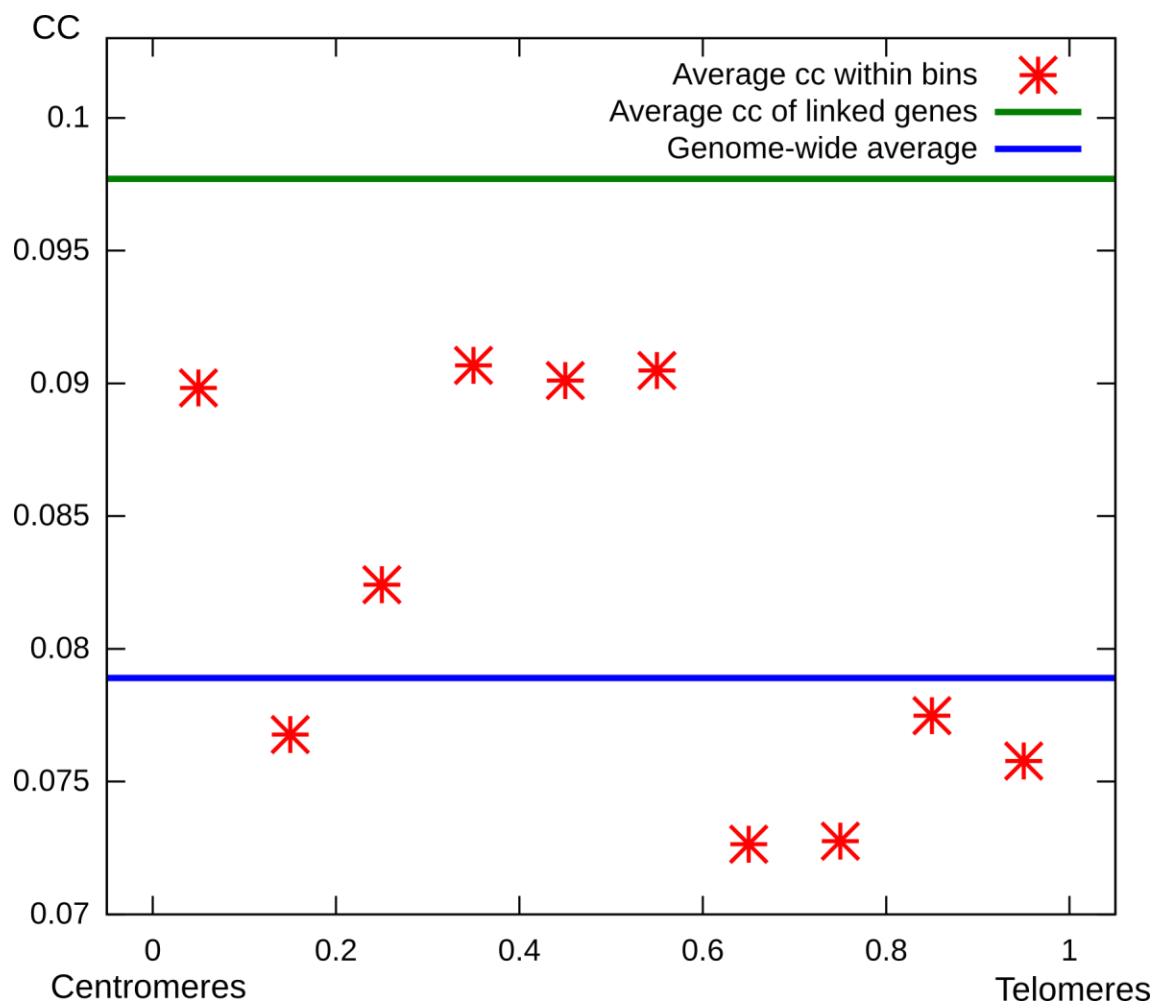

Supplement: Figure S6 — The coexpression of interacting genes cannot be explained by telomere or centromere clustering. Blue solid line: The average correlation of expression profiles for all interchromosomal gene pairs in the genome. Green solid line: The average correlation of expression profiles for pairs of genes associated with DNA interactions measured by 4C. Red points: The average correlation of expression profiles within groups of genes with similar relative position between the centromere and telomere. (PDF) [file pone.0054699.s006.pdf]

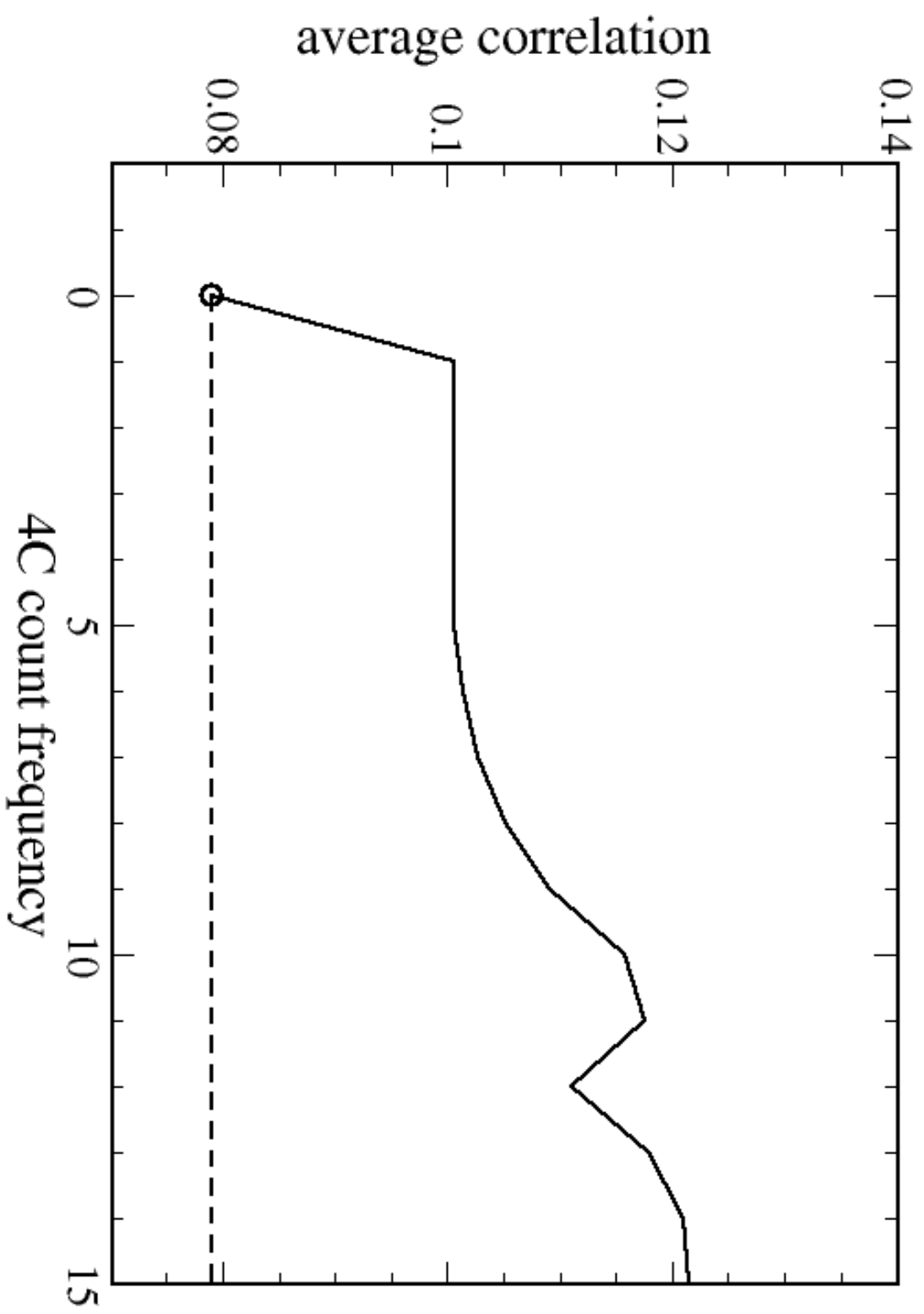

Supplement: Figure S7 — The average correlation between linked genes as a function of the experimental count frequency of the corresponding contacts based on the EcoRI library. Frequency of zero corresponds to all possible pairs of genes (linked and unlinked) and represents the genome wide average for all possible inter-chromosomal pairs of genes. The genome wide average is highlighted here by the circle at the horizontal dashed line for improving the visual comparison. (PDF) [file pone.0054699.s007.pdf]

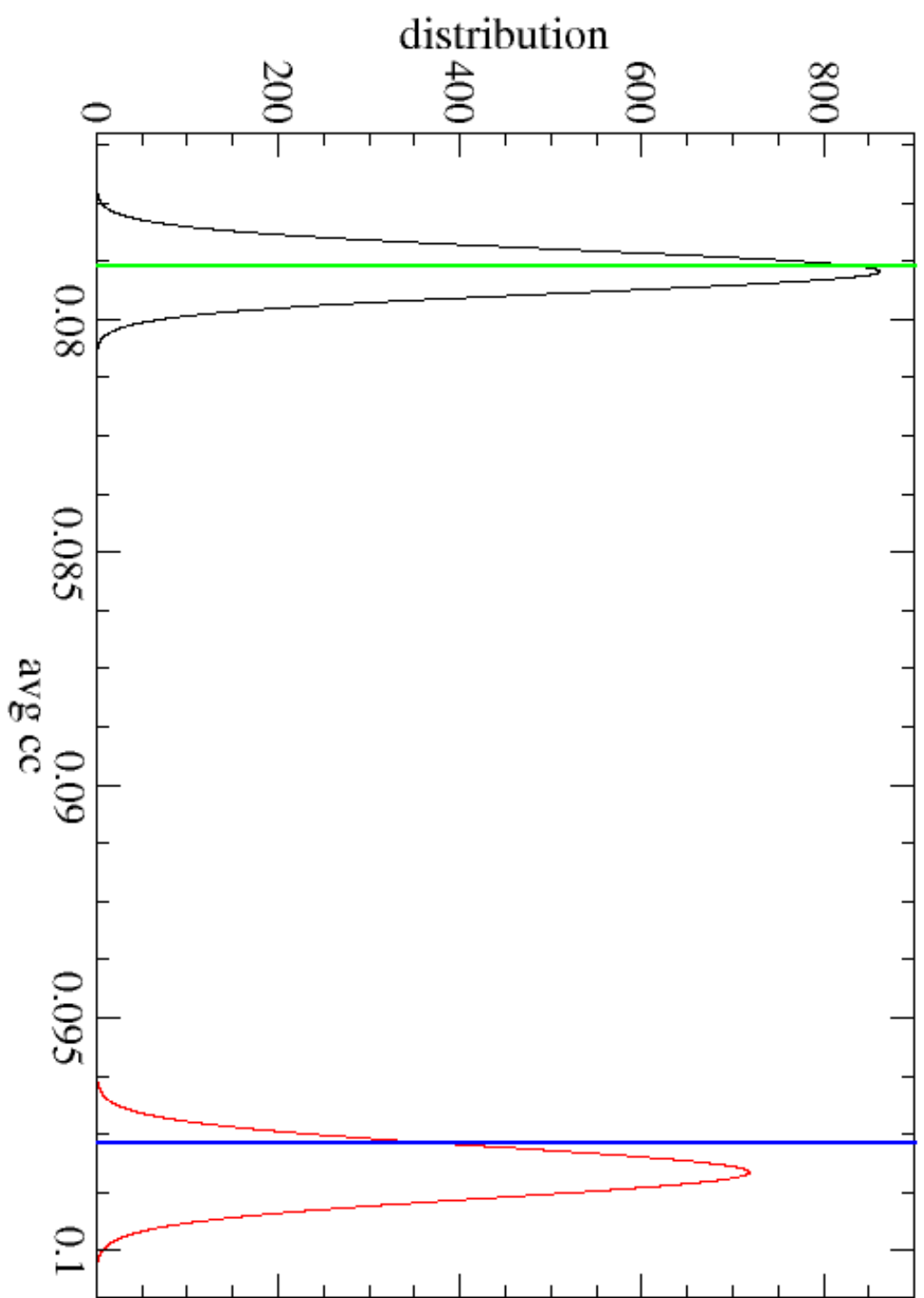

Supplement: Figure S8 — The significance of coexpression of genes associated with interacting loci. Black: The histogram of 30,000 average correlation coefficients within groups of randomly chosen genes, each generated by choosing 240629 pairs of genes from the entire genome. (green line shows the genome average). Red: The histogram of 1000 average correlation coefficients between linked genes, generated by bootstrapping (choosing a random subset of 120300 interactions between linked genes). Blue line shows the average of all interacting genes. (PDF) [file pone.0054699.s008.pdf]
