# Supplementary material for: The 3D Organization of the Yeast Genome Correlates with Co-Expression and Reflects Functional Relations between Genes
Source: PLoS One. 2013 Jan 31;8(1):e54699. doi: 10.1371/journal.pone.0054699 (PMC3561378; doi:10.1371/journal.pone.0054699)
Supplement: Table S1 — A listing of the number observed 4C contacts for all GO-slim terms versus the expected number. The numbers are calculated at different threshold count frequencies. Monte Carlo simulations are used to generate 1000 random samples for each term. The expected number of contacts is determined from the average number of contacts in the 1000 samples and the standard deviation gives the Z-score. (PDF) [file pone.0054699.s009.pdf]

(A) Molecular Function

	Frequency > 4			Frequency > 5			Frequency > 6			Frequency > 7			Frequency > 8			Frequency > 9		
	observed	expected	Z-score	observed	expected	Z-score	observed	expected	Z-score	observed	expected	Z-score	observed	expected	Z-score	observed	expected	Z-score
molecular function	26484	34034	-4	22940	29664	-4	13659	17907	-3	8485	11315	-3	5434	7375	-3	3538	5017	-3
hydrolase activity	4168	2748	4	3688	2396	4	2308	1464	3	1490	926	3	1023	592	3	718	407	3
transferase activity	2585	2001	2	2232	1736	2	1321	1053	1	834	660	1	521	436	0	345	297	0
protein binding	1591	1268	1	1418	1093	1	863	663	1	549	419	1	379	273	1	279	186	1
transporter activity	960	595	3	828	524	2	480	313	2	294	197	1	182	130	1	118	86	1
DNA binding	588	505	0	510	442	0	310	267	0	189	168	0	114	109	0	73	73	0
structural molecule activity	300	501	-2	249	433	-2	143	260	-1	91	163	-1	47	108	-1	29	73	-1
transcription regulator activity	556	439	1	472	384	1	286	232	0	183	146	0	122	95	0	89	65	1
oxidoreductase activity	345	320	0	303	273	0	194	166	0	120	106	0	79	67	0	59	47	0
RNA binding	179	206	0	161	182	0	88	109	0	51	67	0	39	45	0	24	31	0
enzyme regulator activity	183	192	0	168	165	0	117	101	0	69	64	0	43	41	0	24	27	0
ligase activity	216	131	2	197	114	2	124	68	2	79	43	2	51	28	1	29	19	0
protein kinase activity	103	69	1	90	60	1	53	36	1	35	23	1	23	15	0	12	10	0
peptidase activity	72	52	1	60	46	0	38	28	0	23	17	0	19	11	1	16	7	1
lyase activity	33	30	0	27	26	0	18	16	0	9	9	0	8	6	0	6	4	0
helicase activity	48	28	1	46	24	1	35	15	2	18	9	1	15	6	1	12	4	2
lipid binding	18	21	0	14	19	0	9	11	0	6	7	0	4	4	0	2	3	0
nucleotidyltransferase activity	12	18	0	10	16	0	1	9	-1	0	5	-1	0	3	0	0	2	0
isomerase activity	19	13	0	17	11	0	10	7	0	8	4	1	6	2	1	4	2	0
phosphoprotein phosphatase activity	13	10	0	12	8	0	7	5	0	5	3	0	4	2	0	3	1	1
signal transducer activity	16	8	1	14	7	1	7	4	0	7	2	1	6	1	2	4	1	1
motor activity	1	0	0	1	0	0	0	0	0	0	0	0	0	0	0	0	0	0
translation regulator activity	0	0	0	0	0	0	0	0	0	0	0	0	0	0	0	0	0	0

(B) Biological Process

biological process	10854	18099	-5	9233	15698	-5	5367	9500	-5	3285	6001	-4	2039	3928	-4	1273	2675	-4
RNA metabolic process	6454	5115	2	5676	4483	2	3480	2686	2	2268	1721	2	1541	1118	2	1089	756	2
transport	5785	4670	2	5099	4059	2	3104	2440	2	2043	1541	2	1396	1012	2	986	678	2
transcription	2323	1608	3	2047	1407	3	1259	852	2	816	536	2	510	351	2	362	233	2
response to stress	2027	1424	2	1785	1251	2	1132	746	2	736	478	2	505	313	2	364	210	2
protein modification process	1772	1304	2	1561	1140	2	967	692	2	615	438	1	393	285	1	283	195	1
cell cycle	2134	1239	4	1870	1079	4	1170	650	4	716	411	3	459	267	2	299	184	2
DNA metabolic process	936	683	2	839	590	2	504	356	1	317	226	1	200	149	1	140	101	1
response to chemical stimulus	981	663	2	810	584	2	483	352	1	308	224	1	192	146	1	130	96	1
chromosome organization	929	648	2	825	572	2	549	342	2	378	217	2	259	142	2	195	95	3
translation	513	625	0	456	542	0	268	327	0	171	208	0	112	136	0	71	93	0
vesicle-mediated transport	589	538	0	511	476	0	325	284	0	210	182	0	137	118	0	101	81	0
ribosome biogenesis	515	498	0	471	431	0	296	260	0	198	165	0	139	107	0	95	72	0
mitochondrion organization	249	363	-1	229	316	-1	146	190	0	99	121	0	70	78	0	51	53	0
cellular membrane organization	353	333	0	298	289	0	171	175	0	90	111	0	57	72	0	39	50	0
cellular carbohydrate metabolic process	420	320	1	350	280	1	215	166	1	127	105	0	79	69	0	59	46	0
cellular amino acid and derivative metabolic process	376	285	1	331	250	1	198	149	1	100	94	0	58	61	0	34	42	0
signal transduction	356	221	2	314	190	2	198	114	2	138	72	2	95	47	2	66	31	2
cytoskeleton organization	241	217	0	214	187	0	135	113	0	89	72	0	54	46	0	35	31	0
protein complex biogenesis	159	209	-1	144	184	0	91	109	0	72	69	0	52	44	0	45	30	1
cellular lipid metabolic process	252	198	1	223	176	0	138	105	1	81	66	0	56	43	0	45	29	1
cellular protein catabolic process	245	153	2	216	133	2	140	81	2	99	51	2	69	33	2	52	22	2
heterocycle metabolic process	184	152	0	164	130	0	102	79	0	63	50	0	40	32	0	25	22	0
generation of precursor metabolites and energy	128	140	0	102	123	0	61	74	0	41	46	0	24	29	0	16	20	0
cofactor metabolic process	146	127	0	136	110	0	87	66	0	56	42	0	35	27	0	20	19	0
cellular component morphogenesis	255	127	3	199	111	2	119	65	2	68	41	1	48	26	1	33	18	1
meiosis	188	104	2	170	91	2	98	54	2	60	34	1	30	22	0	18	15	0
chromosome segregation	157	88	2	143	76	2	85	46	2	58	29	2	39	20	1	28	13	1
cellular homeostasis	109	84	0	97	76	0	52	45	0	37	28	0	27	18	0	16	12	0
sporulation resulting in formation of a cellular spore	109	73	1	87	62	1	58	39	1	32	24	0	22	16	0	12	10	0
fungal-type cell wall organization	99	72	1	93	62	1	58	37	1	38	24	1	28	16	1	17	10	0
conjugation	67	62	0	57	53	0	28	32	0	17	21	0	11	13	0	9	8	0
cytokinesis	97	59	1	74	53	1	39	31	0	20	20	0	11	13	0	7	8	0
cellular respiration	35	37	0	26	33	0	14	19	0	10	12	0	4	8	0	3	5	0
cell budding	55	33	1	47	29	1	28	17	1	17	11	0	11	7	0	7	5	0
protein folding	42	33	0	37	29	0	22	17	0	12	11	0	7	7	0	6	5	0
cellular aromatic compound metabolic process	32	25	0	28	22	0	20	13	0	10	8	0	2	5	0	0	3	0
vesicle organization	30	22	0	28	18	1	13	11	0	6	7	0	2	4	0	2	3	0
nucleus organization	32	18	1	29	16	1	19	9	1	10	6	0	6	4	0	2	2	0
pseudohyphal growth	18	17	0	17	15	0	13	9	0	8	5	0	4	4	0	1	2	0
vacuole organization	15	16	0	12	14	0	6	8	0	3	5	0	3	3	0	2	2	0
vitamin metabolic process	30	16	1	29	14	1	19	8	1	14	5	2	8	3	1	8	2	2
peroxisome organization	19	15	0	18	13	0	14	8	1	13	5	2	8	3	1	6	2	1
transposition	17	8	1	13	8	0	10	5	1	8	2	2	6	2	1	3	1	1

(C) Cellular Component

cytoplasm	57286	52552	2	49763	45855	1	29789	27691	1	18760	17554	1	12332	11445	1	8449	7753	1
nucleus	19899	15564	4	17566	13579	3	10826	8235	3	7017	5184	3	4625	3385	3	3200	2298	2
cellular component	5975	10883	-5	5091	9511	-5	3008	5720	-5	1924	3635	-4	1211	2372	-3	795	1603	-3
membrane	7578	5646	3	6618	4893	3	4066	2967	3	2625	1884	2	1798	1227	2	1255	824	2
mitochondrion	5209	4915	0	4476	4270	0	2690	2580	0	1682	1628	0	1129	1062	0	785	727	0
endoplasmic reticulum	695	587	0	616	517	0	379	312	0	252	197	1	168	130	0	103	88	0
ribosome	285	505	-2	236	439	-2	138	265	-2	78	170	-1	45	109	-1	27	74	-1
chromosome	738	490	2	673	426	2	408	259	2	278	161	2	177	107	2	131	73	2
endomembrane system	525	459	0	461	402	0	281	238	0	183	152	0	122	100	0	82	67	0
mitochondrial envelope	359	415	0	311	359	0	202	217	0	129	137	0	99	89	0	67	60	0
plasma membrane	669	355	4	579	305	3	373	183	3	261	118	4	180	77	3	124	52	3
nucleolus	380	246	2	352	213	2	232	132	2	160	82	2	115	53	3	86	36	3
site of polarized growth	371	217	2	328	196	2	207	118	2	117	73	1	77	48	1	53	31	1
vacuole	242	186	1	198	165	0	108	100	0	68	63	0	45	39	0	33	27	0
cytoskeleton	266	184	1	222	162	1	140	99	1	76	62	0	49	40	0	32	27	0
membrane fraction	194	171	0	165	149	0	111	89	0	67	56	0	43	37	0	27	24	0
cellular bud	230	143	2	203	124	2	130	75	2	67	48	1	47	29	1	26	20	0
Golgi apparatus	138	132	0	128	116	0	87	69	0	67	44	1	47	28	1	36	18	1
cell cortex	124	60	3	99	54	2	58	32	1	26	20	0	17	13	0	10	9	0
cytoplasmic membrane-bounded vesicle	55	47	0	46	41	0	30	25	0	24	15	1	17	9	1	14	6	1
cell wall	37	42	0	28	36	0	19	21	0	9	13	0	4	8	0	2	6	0
microtubule organizing center	20	22	0	16	18	0	8	11	0	2	7	0	1	4	0	0	3	0
peroxisome	12	16	0	10	14	0	7	8	0	4	5	0	3	3	0	1	2	0
extracellular region	1	3	0	1	2	0	0	1	0	0	0	0	0	0	0	0	0	0
