# Supplementary material for: The 3D Organization of the Yeast Genome Correlates with Co-Expression and Reflects Functional Relations between Genes
Source: PLoS One. 2013 Jan 31;8(1):e54699. doi: 10.1371/journal.pone.0054699 (PMC3561378; doi:10.1371/journal.pone.0054699)
Supplement: Table S2 — This table lists the GEO accession numbers for 1496 gene expression microarray samples used in this work. (PDF) [file pone.0054699.s010.pdf]

GEO Expression Data

The following is a listing of the GEO accession numbers for 1496 gene expression microarray samples used in this work:

GSM6711	GSM6712	GSM6713	GSM6715	GSM6717	GSM6726
GSM6728	GSM6729	GSM6730	GSM6731	GSM6732	GSM6219
GSM6220	GSM6221	GSM6222	GSM6223	GSM6224	GSM6225
GSM6226	GSM7490	GSM7491	GSM7492	GSM7493	GSM7494
GSM7495	GSM7496	GSM12761	GSM12633	GSM12754	GSM12755
GSM12756	GSM24746	GSM29920	GSM29921	GSM29922	GSM29924
GSM29926	GSM29927	GSM29929	GSM29930	GSM29932	GSM29934
GSM29936	GSM29937	GSM29939	GSM29940	GSM29942	GSM29943
GSM29945	GSM29946	GSM29948	GSM29949	GSM29951	GSM27837
GSM27838	GSM27839	GSM27840	GSM27841	GSM27842	GSM27843
GSM27844	GSM27845	GSM27846	GSM27847	GSM27848	GSM27849
GSM27850	GSM27851	GSM27852	GSM27853	GSM27854	GSM31653
GSM31654	GSM31655	GSM31656	GSM31657	GSM31658	GSM31659
GSM31660	GSM31661	GSM31662	GSM31663	GSM31664	GSM29073
GSM29077	GSM29080	GSM29083	GSM29086	GSM29089	GSM29094
GSM29098	GSM29101	GSM29104	GSM29105	GSM29106	GSM29107
GSM29108	GSM29109	GSM29110	GSM34633	GSM34634	GSM34635
GSM34678	GSM34679	GSM34680	GSM34681	GSM34682	GSM34683
GSM34684	GSM34685	GSM34686	GSM34687	GSM34688	GSM34689
GSM34690	GSM34691	GSM34692	GSM34693	GSM34694	GSM34695
GSM34696	GSM34697	GSM34698	GSM34699	GSM34700	GSM34701
GSM34752	GSM34753	GSM34754	GSM34755	GSM34756	GSM34757
GSM34758	GSM34759	GSM34760	GSM34761	GSM34762	GSM34763
GSM34764	GSM34765	GSM34766	GSM12927	GSM12928	GSM12929
GSM12930	GSM12931	GSM12932	GSM35294	GSM35295	GSM35296
GSM35297	GSM35298	GSM35299	GSM35300	GSM35301	GSM35302
GSM35303	GSM35304	GSM35305	GSM35306	GSM35307	GSM35308
GSM35309	GSM35310	GSM35311	GSM35312	GSM35313	GSM35314
GSM35315	GSM35316	GSM35317	GSM35318	GSM35319	GSM35320
GSM35321	GSM34889	GSM34890	GSM34891	GSM34892	GSM35972
GSM35973	GSM35974	GSM35975	GSM35976	GSM35977	GSM15273
GSM15275	GSM15277	GSM15279	GSM15281	GSM15283	GSM37612
GSM37613	GSM37614	GSM37615	GSM37616	GSM37617	GSM37618
GSM37619	GSM37620	GSM37621	GSM37622	GSM37623	GSM37903
GSM37904	GSM37905	GSM37906	GSM37907	GSM37908	GSM40714
GSM40715	GSM40716	GSM40717	GSM40718	GSM40719	GSM40720
GSM40721	GSM40722	GSM40723	GSM40724	GSM40725	GSM40726
GSM40727	GSM40728	GSM40729	GSM40730	GSM40731	GSM40732
GSM40733	GSM40734	GSM40735	GSM40736	GSM40737	GSM43898
GSM43899	GSM43900	GSM43901	GSM43902	GSM43903	GSM43904
GSM43905	GSM43906	GSM43907	GSM43908	GSM43909	GSM49659
GSM49660	GSM49662	GSM49663	GSM49664	GSM49665	GSM48131
GSM48132	GSM48133	GSM48134	GSM48135	GSM48136	GSM48137
GSM48138	GSM48139	GSM48140	GSM48141	GSM48142	GSM48143
GSM48144	GSM48145	GSM48146	GSM48147	GSM48148	GSM48149

GSM48150	GSM48151	GSM48152	GSM48153	GSM48154	GSM48155
GSM48156	GSM48157	GSM48158	GSM48159	GSM48160	GSM48161
GSM48162	GSM43865	GSM43866	GSM43867	GSM43868	GSM43869
GSM43897	GSM50009	GSM50010	GSM50011	GSM50012	GSM51901
GSM51902	GSM51903	GSM51904	GSM51905	GSM51906	GSM51907
GSM51908	GSM51909	GSM21640	GSM21641	GSM21642	GSM21643
GSM21644	GSM21645	GSM21646	GSM21647	GSM21648	GSM21649
GSM21650	GSM21651	GSM21652	GSM21653	GSM21654	GSM21655
GSM21656	GSM21657	GSM21658	GSM21659	GSM21660	GSM21661
GSM21662	GSM21663	GSM21664	GSM21665	GSM21666	GSM21667
GSM21668	GSM21669	GSM21670	GSM21671	GSM21672	GSM21673
GSM21674	GSM21675	GSM21676	GSM21677	GSM21678	GSM21679
GSM21680	GSM21681	GSM21682	GSM21683	GSM21684	GSM21685
GSM21686	GSM21687	GSM21688	GSM21689	GSM21690	GSM21691
GSM21692	GSM21693	GSM21694	GSM21695	GSM21696	GSM21697
GSM21698	GSM21699	GSM21700	GSM21701	GSM21702	GSM21703
GSM21704	GSM21705	GSM71925	GSM71926	GSM71927	GSM71928
GSM71929	GSM71930	GSM71931	GSM71932	GSM71933	GSM71934
GSM71935	GSM45194	GSM45279	GSM45281	GSM45282	GSM45283
GSM45284	GSM77298	GSM77299	GSM77300	GSM77301	GSM77302
GSM77303	GSM77304	GSM77305	GSM77306	GSM77307	GSM77308
GSM77309	GSM77310	GSM77311	GSM77312	GSM77313	GSM77314
GSM77315	GSM77316	GSM77317	GSM77318	GSM77319	GSM77320
GSM77321	GSM77322	GSM77323	GSM77324	GSM77325	GSM77326
GSM77327	GSM77328	GSM77329	GSM77330	GSM77331	GSM77332
GSM77333	GSM87661	GSM87662	GSM87663	GSM87664	GSM87665
GSM87666	GSM87667	GSM87668	GSM87669	GSM87670	GSM87671
GSM87672	GSM87673	GSM87674	GSM87675	GSM87676	GSM75830
GSM75831	GSM75832	GSM94403	GSM94538	GSM94539	GSM94540
GSM94555	GSM94587	GSM94589	GSM94590	GSM94591	GSM94592
GSM94593	GSM94594	GSM94595	GSM94596	GSM88277	GSM88278
GSM88279	GSM88280	GSM88281	GSM65640	GSM66919	GSM66920
GSM66921	GSM66922	GSM66923	GSM86746	GSM86747	GSM86748
GSM86749	GSM86750	GSM86751	GSM86752	GSM86753	GSM86755
GSM86756	GSM86757	GSM86758	GSM86759	GSM86761	GSM86762
GSM86763	GSM86764	GSM86767	GSM86768	GSM86769	GSM108368
GSM108369	GSM108370	GSM108392	GSM108393	GSM108394	GSM98594
GSM98595	GSM98596	GSM98597	GSM98598	GSM98599	GSM105139
GSM105143	GSM105145	GSM105146	GSM60930	GSM60931	GSM60932
GSM60933	GSM60934	GSM60935	GSM119751	GSM119752	GSM119753
GSM119754	GSM119755	GSM119756	GSM119757	GSM119758	GSM105333
GSM105334	GSM105335	GSM105336	GSM105337	GSM105338	GSM105339
GSM105340	GSM105341	GSM105342	GSM105343	GSM105344	GSM67545
GSM67546	GSM67547	GSM67548	GSM67549	GSM67550	GSM67551
GSM67552	GSM67553	GSM67554	GSM67555	GSM67556	GSM67557
GSM67558	GSM67559	GSM67560	GSM67561	GSM67562	GSM67563
GSM67564	GSM67565	GSM67566	GSM67567	GSM67568	GSM67569
GSM67570	GSM67571	GSM67572	GSM67573	GSM67574	GSM67575
GSM67576	GSM67577	GSM67578	GSM67579	GSM67580	GSM67581
GSM67582	GSM67583	GSM67584	GSM67585	GSM67586	GSM67587
GSM67588	GSM67589	GSM67590	GSM67591	GSM67592	GSM67593
GSM67594	GSM67595	GSM67596	GSM67597	GSM67598	GSM67599
GSM67600	GSM67601	GSM67602	GSM67603	GSM67604	GSM67605
GSM67606	GSM67607	GSM67608	GSM67609	GSM67610	GSM67611

GSM67612	GSM67613	GSM67614	GSM67615	GSM67616	GSM67617
GSM67618	GSM67619	GSM67620	GSM67621	GSM67622	GSM67623
GSM67624	GSM67625	GSM67626	GSM67627	GSM67628	GSM67629
GSM67630	GSM67631	GSM67632	GSM67633	GSM67634	GSM67635
GSM67636	GSM67637	GSM67638	GSM67639	GSM67640	GSM120128
GSM120129	GSM120130	GSM120131	GSM120132	GSM120133	GSM120134
GSM120135	GSM120136	GSM120138	GSM120139	GSM120143	GSM120144
GSM120145	GSM140786	GSM140800	GSM140801	GSM140802	GSM140803
GSM140804	GSM140805	GSM140808	GSM140809	GSM140810	GSM140811
GSM140812	GSM153907	GSM153908	GSM153909	GSM153910	GSM153911
GSM153912	GSM153913	GSM153914	GSM153915	GSM153916	GSM154157
GSM154623	GSM154627	GSM154628	GSM154629	GSM154631	GSM143071
GSM143073	GSM143076	GSM143077	GSM143079	GSM143081	GSM143082
GSM143083	GSM143084	GSM143085	GSM143088	GSM143089	GSM142982
GSM142983	GSM142984	GSM142985	GSM142986	GSM142987	GSM120137
GSM120140	GSM120141	GSM120142	GSM137676	GSM137677	GSM137678
GSM137679	GSM137680	GSM137681	GSM137682	GSM137683	GSM137684
GSM137685	GSM137686	GSM137687	GSM147746	GSM147747	GSM147748
GSM147749	GSM147750	GSM147751	GSM147753	GSM147754	GSM147755
GSM147759	GSM147761	GSM147762	GSM137823	GSM137824	GSM137825
GSM137826	GSM137827	GSM137828	GSM137829	GSM137830	GSM137831
GSM137832	GSM137833	GSM137834	GSM137835	GSM137836	GSM137837
GSM137838	GSM137839	GSM137840	GSM137841	GSM137842	GSM137843
GSM137844	GSM137845	GSM137846	GSM137847	GSM137848	GSM137849
GSM137850	GSM137851	GSM137852	GSM137853	GSM137854	GSM137855
GSM137856	GSM137857	GSM137858	GSM137859	GSM137860	GSM137861
GSM137862	GSM137863	GSM137864	GSM137865	GSM137866	GSM137867
GSM137868	GSM137869	GSM137870	GSM137871	GSM137872	GSM137873
GSM137874	GSM137875	GSM137876	GSM137877	GSM137878	GSM137879
GSM137880	GSM137881	GSM137882	GSM137883	GSM137884	GSM137885
GSM137886	GSM137887	GSM137888	GSM137889	GSM137890	GSM137891
GSM137892	GSM153627	GSM153628	GSM153629	GSM153630	GSM153631
GSM153632	GSM153633	GSM153634	GSM206381	GSM206382	GSM206383
GSM206384	GSM206385	GSM206386	GSM206387	GSM206388	GSM206389
GSM206390	GSM206391	GSM206392	GSM206393	GSM206394	GSM206395
GSM207569	GSM207570	GSM207571	GSM207572	GSM207573	GSM207574
GSM207575	GSM207576	GSM146301	GSM146302	GSM146303	GSM146304
GSM146305	GSM146306	GSM146316	GSM146317	GSM146318	GSM146319
GSM146320	GSM146321	GSM146322	GSM146323	GSM146324	GSM146325
GSM146326	GSM146327	GSM146328	GSM146329	GSM225399	GSM225400
GSM225401	GSM225402	GSM225403	GSM225404	GSM225405	GSM225406
GSM225407	GSM225461	GSM225463	GSM225466	GSM225467	GSM225468
GSM225469	GSM225482	GSM225514	GSM225515	GSM225516	GSM225517
GSM225519	GSM225520	GSM225521	GSM216549	GSM216550	GSM216551
GSM216552	GSM176869	GSM176894	GSM176895	GSM176896	GSM176898
GSM176900	GSM176901	GSM176902	GSM176903	GSM172916	GSM172917
GSM172918	GSM172919	GSM172920	GSM172921	GSM172922	GSM172923
GSM198364	GSM198365	GSM198366	GSM198367	GSM198368	GSM198369
GSM200685	GSM200686	GSM200687	GSM200688	GSM200689	GSM200690
GSM200691	GSM200692	GSM200693	GSM234643	GSM234644	GSM234645
GSM184944	GSM184945	GSM184946	GSM184947	GSM184948	GSM184949
GSM184950	GSM184951	GSM184952	GSM184953	GSM184954	GSM184955
GSM184956	GSM184957	GSM184958	GSM184959	GSM184960	GSM184961
GSM184962	GSM184963	GSM184964	GSM184965	GSM184966	GSM184967

GSM184968	GSM184969	GSM184970	GSM184971	GSM184972	GSM184973
GSM184974	GSM184975	GSM184976	GSM184977	GSM184978	GSM184979
GSM184980	GSM184981	GSM184982	GSM184983	GSM184984	GSM184985
GSM184986	GSM184987	GSM184988	GSM184989	GSM184990	GSM184991
GSM239654	GSM239655	GSM239656	GSM239657	GSM239658	GSM239659
GSM239660	GSM239661	GSM240641	GSM240642	GSM240643	GSM240644
GSM240645	GSM240646	GSM240647	GSM240648	GSM240649	GSM240650
GSM240651	GSM240652	GSM240653	GSM240654	GSM240655	GSM240656
GSM240657	GSM240658	GSM240659	GSM240660	GSM240661	GSM240662
GSM240663	GSM240664	GSM240665	GSM240666	GSM240667	GSM240668
GSM240669	GSM240670	GSM240671	GSM240672	GSM240673	GSM240674
GSM240675	GSM240676	GSM240677	GSM240678	GSM240679	GSM240680
GSM240713	GSM240714	GSM240715	GSM240716	GSM240717	GSM240718
GSM240719	GSM240720	GSM217395	GSM217616	GSM217617	GSM217618
GSM217621	GSM217633	GSM217634	GSM217635	GSM217636	GSM217637
GSM217638	GSM217639	GSM217640	GSM217641	GSM217642	GSM217643
GSM217745	GSM217746	GSM217747	GSM217748	GSM217749	GSM217750
GSM217751	GSM217752	GSM241146	GSM241147	GSM241148	GSM241149
GSM241150	GSM241151	GSM241152	GSM241153	GSM118581	GSM118582
GSM118583	GSM118584	GSM118585	GSM118586	GSM118587	GSM118588
GSM118589	GSM118590	GSM118591	GSM118592	GSM29912	GSM29914
GSM29917	GSM147756	GSM147757	GSM147758	GSM242654	GSM242655
GSM242660	GSM242661	GSM242662	GSM242665	GSM236941	GSM236942
GSM236943	GSM236944	GSM236945	GSM236946	GSM236947	GSM236948
GSM236949	GSM236950	GSM236951	GSM236952	GSM236953	GSM236954
GSM236955	GSM236956	GSM236957	GSM236958	GSM189732	GSM189733
GSM189734	GSM189735	GSM189736	GSM189737	GSM189738	GSM189739
GSM189740	GSM189741	GSM189742	GSM189743	GSM254979	GSM254980
GSM254981	GSM254982	GSM254768	GSM254769	GSM254770	GSM254771
GSM254772	GSM254773	GSM266723	GSM266724	GSM266725	GSM266726
GSM266728	GSM266729	GSM211831	GSM211832	GSM211833	GSM211834
GSM211835	GSM211836	GSM211837	GSM211838	GSM211839	GSM211840
GSM211841	GSM211842	GSM211843	GSM211844	GSM211845	GSM211846
GSM211847	GSM211848	GSM211849	GSM211850	GSM211851	GSM137497
GSM137498	GSM137675	GSM209972	GSM209973	GSM209974	GSM209975
GSM209976	GSM209977	GSM209978	GSM209979	GSM209980	GSM209981
GSM209982	GSM209983	GSM209984	GSM209985	GSM243561	GSM243562
GSM243563	GSM243564	GSM243565	GSM243566	GSM243567	GSM243568
GSM243569	GSM243570	GSM243571	GSM243572	GSM243573	GSM243574
GSM243575	GSM243631	GSM243632	GSM243633	GSM243634	GSM243635
GSM243636	GSM243637	GSM243638	GSM243639	GSM243640	GSM243641
GSM202390	GSM202391	GSM202392	GSM202393	GSM202394	GSM202395
GSM202396	GSM202397	GSM202398	GSM202399	GSM225566	GSM225571
GSM225577	GSM225579	GSM225583	GSM138684	GSM138685	GSM138686
GSM138687	GSM138688	GSM138689	GSM138690	GSM138691	GSM138692
GSM138693	GSM283150	GSM283151	GSM283152	GSM283153	GSM283154
GSM283155	GSM283156	GSM283157	GSM283158	GSM254433	GSM254434
GSM254435	GSM254436	GSM254438	GSM254443	GSM254444	GSM254445
GSM254446	GSM206793	GSM206794	GSM206795	GSM206796	GSM206797
GSM206798	GSM320738	GSM320760	GSM320761	GSM320762	GSM322926
GSM322927	GSM322936	GSM323150	GSM323151	GSM323152	GSM323153
GSM323155	GSM323156	GSM323158	GSM323159	GSM323160	GSM323161
GSM323162	GSM323163	GSM304520	GSM304521	GSM304531	GSM304532
GSM306018	GSM306019	GSM306020	GSM306021	GSM306022	GSM306023

GSM306024	GSM306025	GSM306026	GSM306027	GSM306028	GSM306029
GSM346651	GSM346652	GSM346653	GSM346654	GSM265821	GSM265822
GSM265823	GSM265824	GSM265825	GSM265826	GSM265827	GSM265828
GSM265829	GSM265830	GSM265831	GSM265832	GSM265833	GSM265834
GSM265835	GSM265836	GSM265837	GSM265838	GSM265839	GSM265840
GSM265841	GSM265842	GSM265843	GSM265844	GSM265845	GSM350771
GSM350772	GSM277242	GSM277243	GSM277244	GSM277245	GSM277250
GSM277251	GSM283692	GSM283693	GSM283694	GSM283695	GSM283696
GSM283697	GSM283698	GSM283699	GSM283700	GSM283868	GSM283869
GSM283870	GSM283871	GSM283872	GSM283873	GSM283897	GSM283898
GSM283899	GSM283900	GSM283901	GSM283903	GSM284058	GSM284059
GSM284060	GSM284061	GSM290691	GSM290692	GSM290744	GSM290745
GSM290746	GSM291100	GSM291103	GSM291104	GSM291105	GSM291106
GSM291107	GSM291340	GSM291341	GSM291342	GSM291343	GSM291344
GSM291345	GSM291346	GSM291347	GSM291348	GSM291349	GSM291350
GSM291351	GSM291418	GSM291419	GSM291420	GSM291431	GSM291432
GSM291448	GSM291449	GSM296157	GSM296158	GSM296159	GSM317639
GSM317647	GSM317837	GSM317838	GSM317839	GSM317840	GSM253508
GSM253509	GSM253510	GSM253511	GSM253512	GSM253513	GSM253514
GSM253515	GSM253516	GSM253517	GSM253518	GSM253519	GSM253520
GSM253521	GSM253522	GSM253523	GSM253524	GSM253525	GSM253526
GSM253527	GSM253528	GSM253529	GSM299685	GSM299686	GSM299687
GSM299688	GSM299689	GSM299690	GSM299697	GSM299698	GSM299774
GSM299775	GSM299776	GSM299777	GSM313535	GSM313536	GSM313537
GSM313538	GSM313539	GSM313540	GSM215115	GSM215116	GSM215117
GSM215119	GSM215121	GSM215122	GSM215129	GSM215130	GSM381326
GSM381327	GSM381328	GSM381329	GSM381330	GSM381331	GSM381332
GSM381333	GSM381334	GSM381335	GSM381336	GSM381337	GSM381338
GSM387533	GSM387534	GSM387535	GSM387536	GSM387537	GSM387538
GSM387539	GSM387540	GSM387541	GSM387542	GSM387543	GSM387544
GSM451048	GSM451049	GSM451050	GSM451051	GSM453346	GSM453347
GSM453348	GSM453349	GSM453350	GSM440152	GSM440153	GSM440154
GSM440155	GSM440156	GSM440195	GSM440196	GSM440197	GSM452841
GSM452842	GSM452843	GSM452844	GSM452845	GSM452846	GSM452847
GSM452848	GSM452849	GSM452850	GSM452851	GSM452852	GSM452853
GSM452854	GSM452855	GSM452856	GSM452857	GSM452858	GSM452859
GSM452860	GSM452861	GSM452862	GSM452863	GSM452864	GSM452865
GSM452866	GSM452867	GSM452868	GSM452869	GSM452870	GSM452871
GSM452872	GSM452873	GSM452874	GSM452875	GSM452876	GSM452877
GSM452878	GSM452879	GSM452880	GSM452881	GSM452882	GSM377493
GSM377494	GSM377495	GSM377496	GSM377497	GSM377498	GSM377499
GSM474474	GSM474475	GSM474864	GSM474865	GSM474866	GSM474867
