# Supplementary material for: The 3D Organization of the Yeast Genome Correlates with Co-Expression and Reflects Functional Relations between Genes
Source: PLoS One. 2013 Jan 31;8(1):e54699. doi: 10.1371/journal.pone.0054699 (PMC3561378; doi:10.1371/journal.pone.0054699)
Supplement: File S1 — Contact networks for GO-slim terms. The figures show the contact networks (frequency >5) for each of the Go-slim terms. The number of links per gene is shown below each figure. (PDF) [file pone.0054699.s011.pdf]

Contact networks for GO-slim terms

The figures below show the contact networks (frequency > 5) for each of the Go-slim terms. The number of links per gene is shown below each figure.

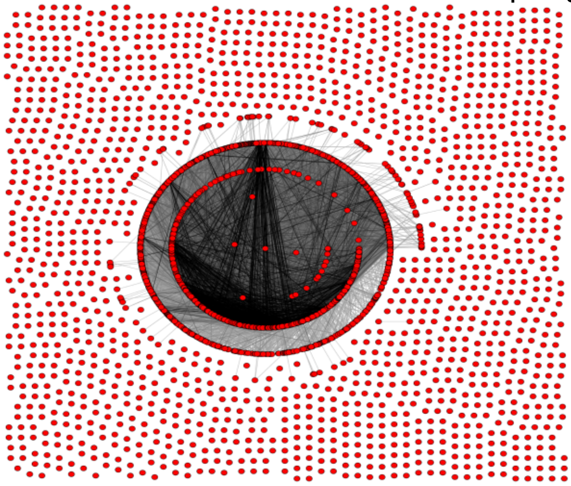

cytoplasm 49763 / 3401

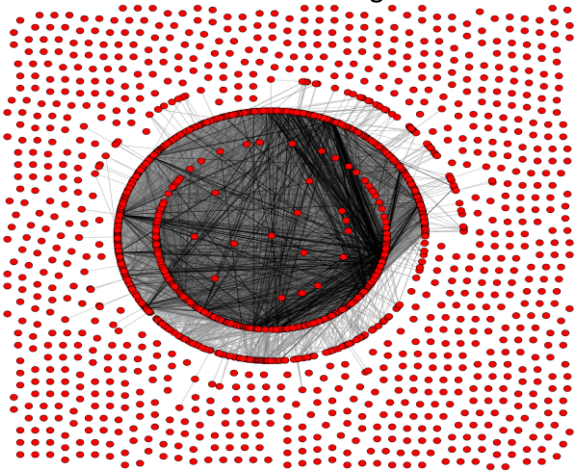

nucleus 17566 / 1852

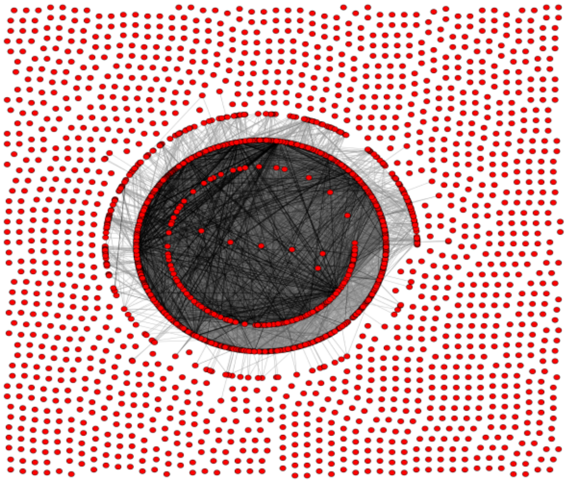

molecular function 22940 / 2734

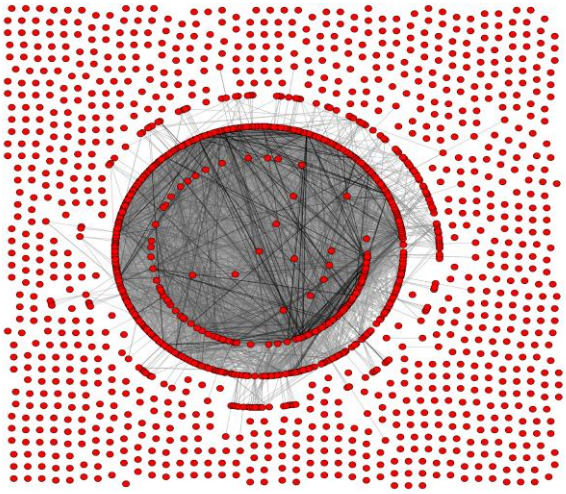

cellular component 5091 / 1548

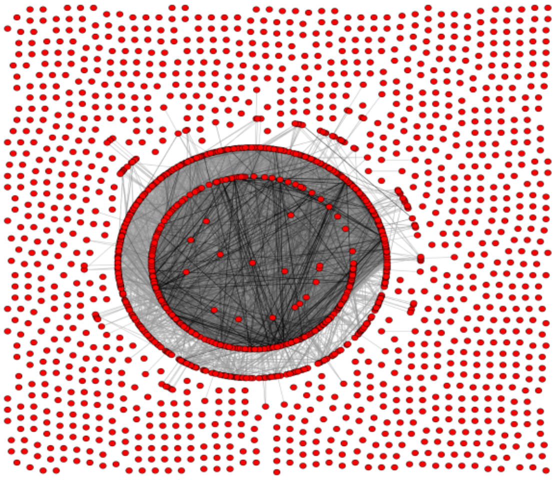

biological process 9233 / 1992

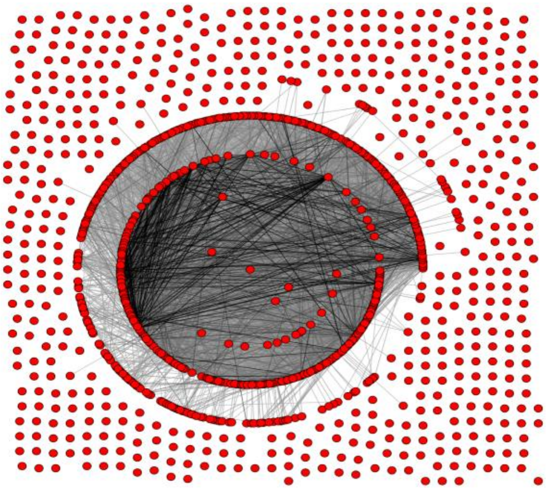

membrane 6618 / 1114

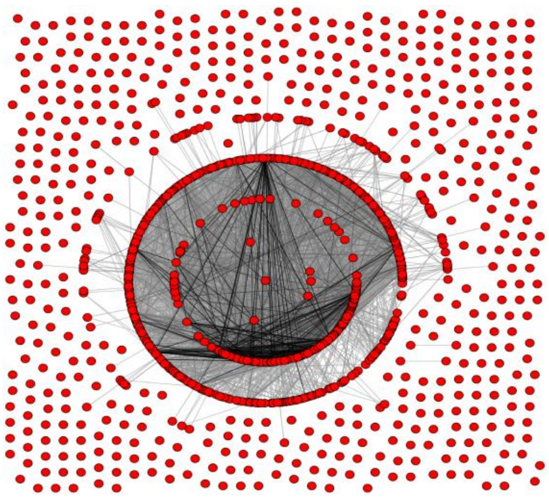

RNA metabolic process 5676 / 1063

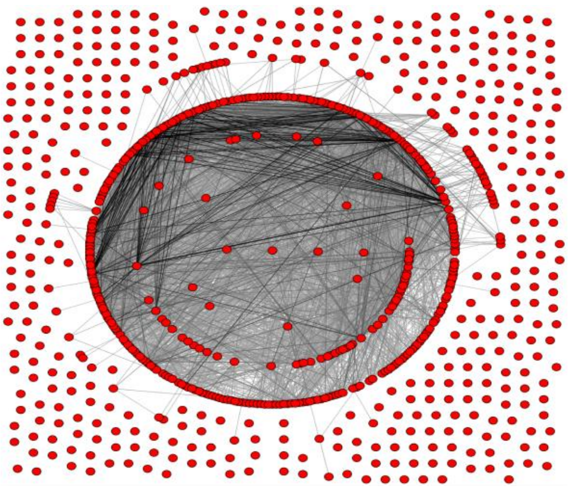

hydrolase activity 3688 / 779

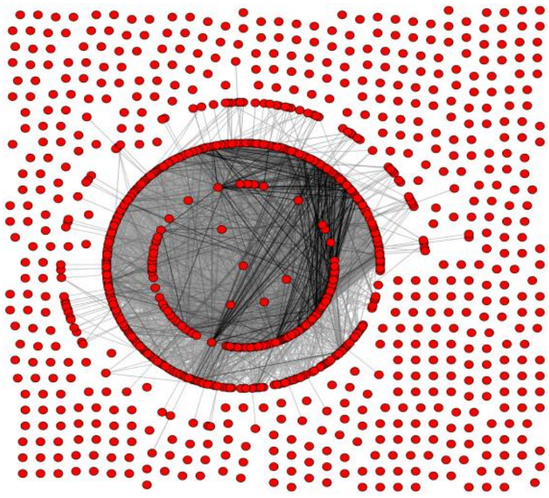

mitochondrion 4476 / 1039

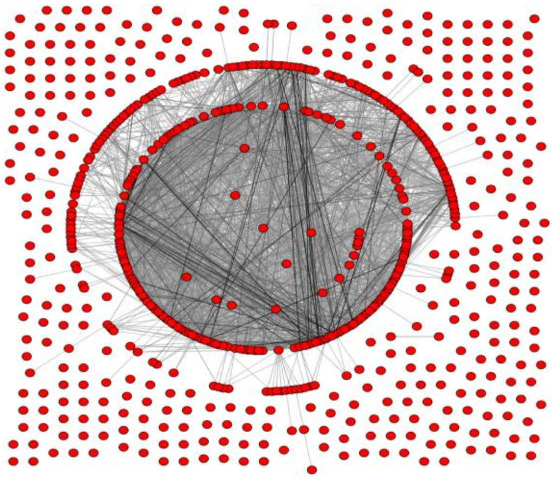

transferase activity 2232 / 662

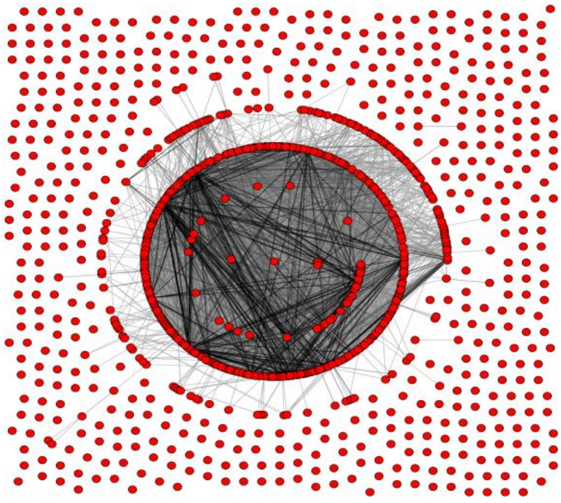

transport 5099 / 1010

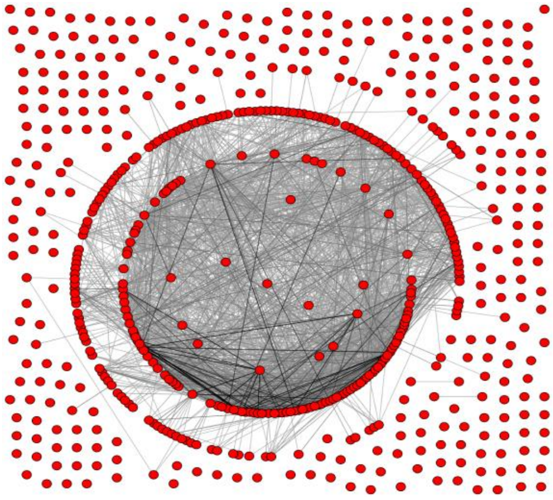

transcription 2047 / 596

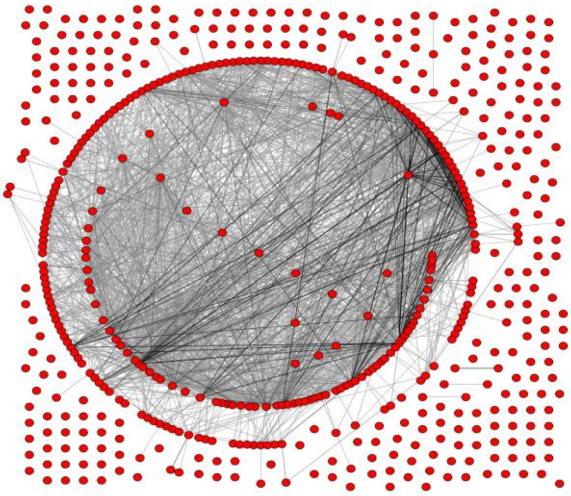

response to stress 1785 / 561

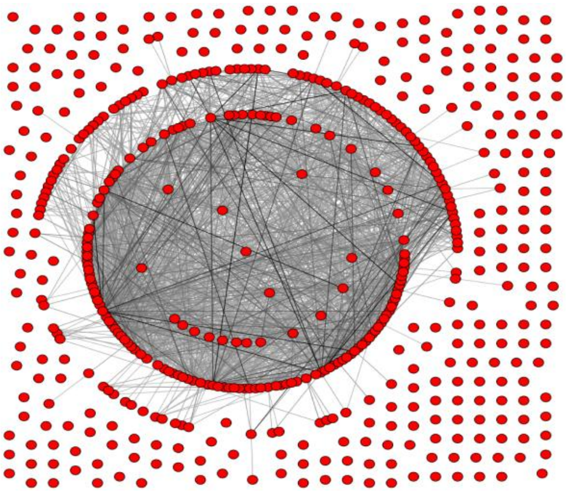

cell cycle 1870 / 521

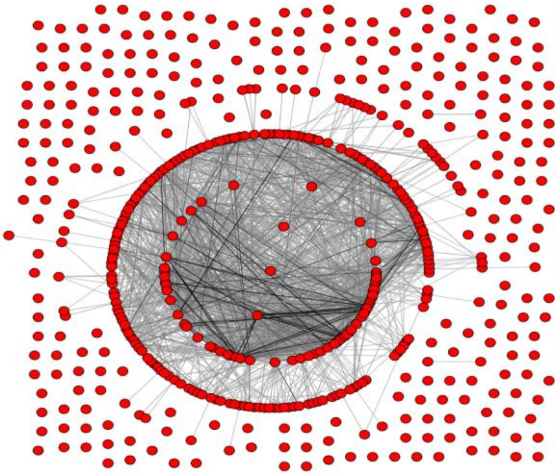

protein modification process 1561 / 537

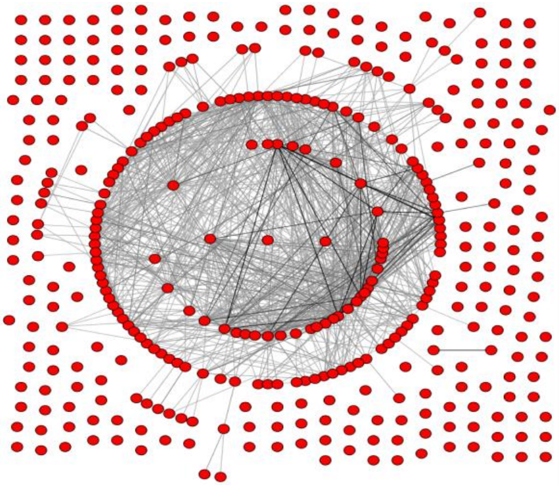

DNA metabolic process 839 / 388

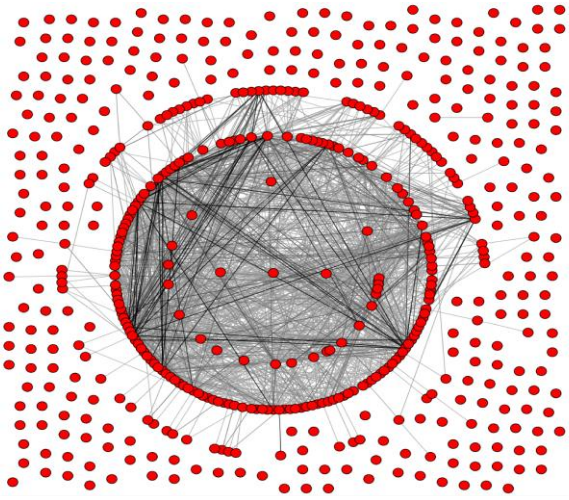

protein binding 1418 / 527

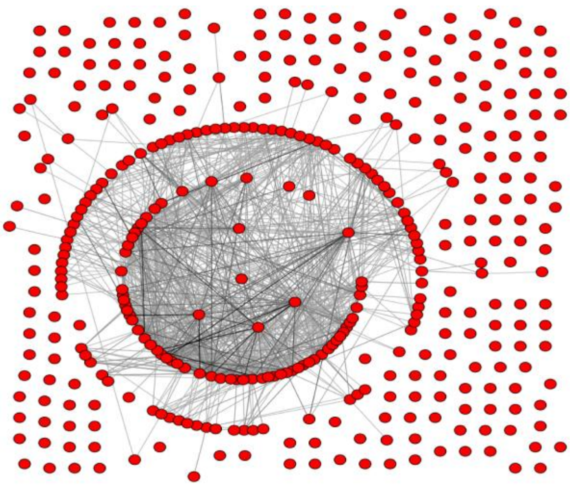

response to chemical stimulus 810 / 383

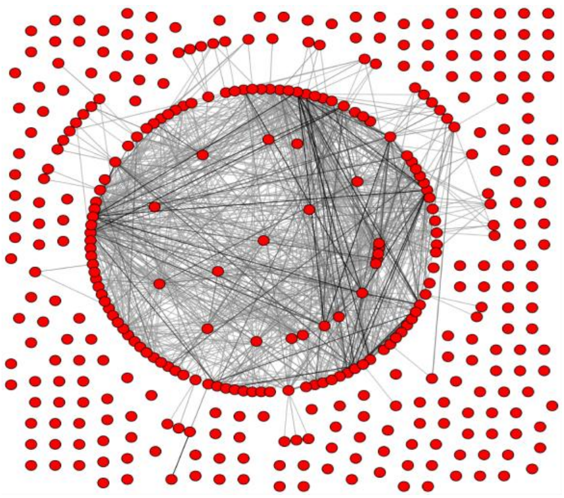

chromosome organization 825 / 379

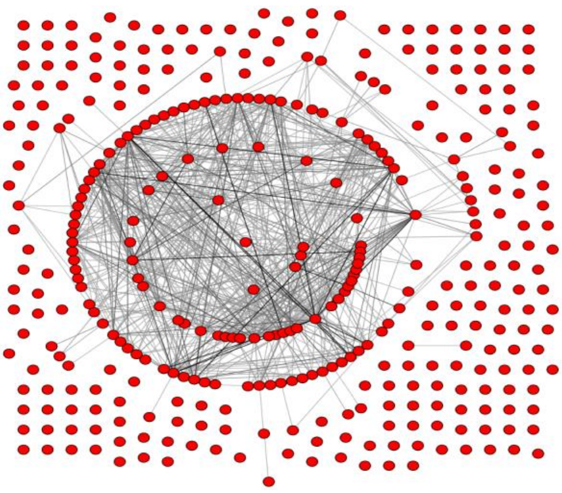

endoplasmic reticulum 616 / 362

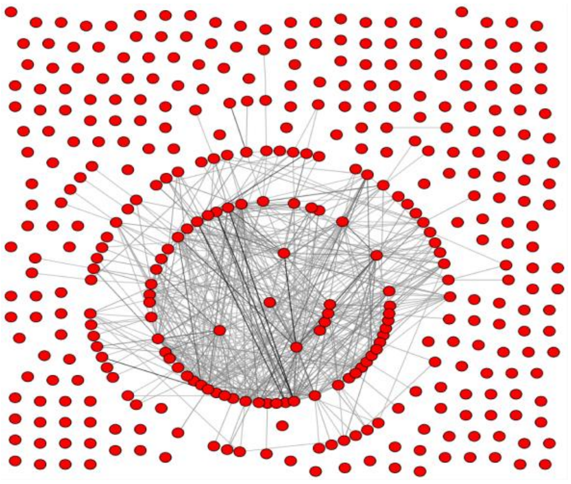

translation 456 / 371

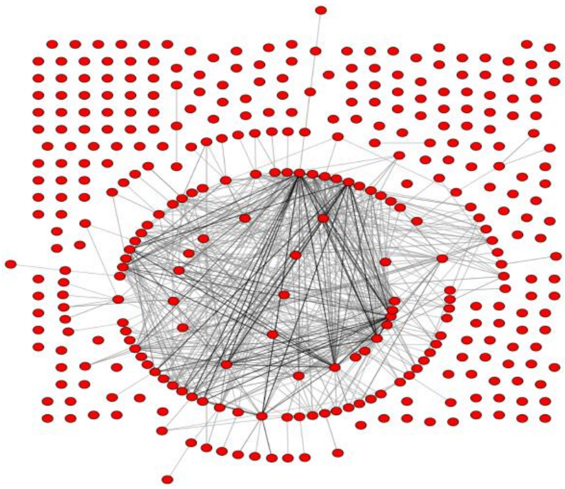

vesicle-mediated transport 511 / 347

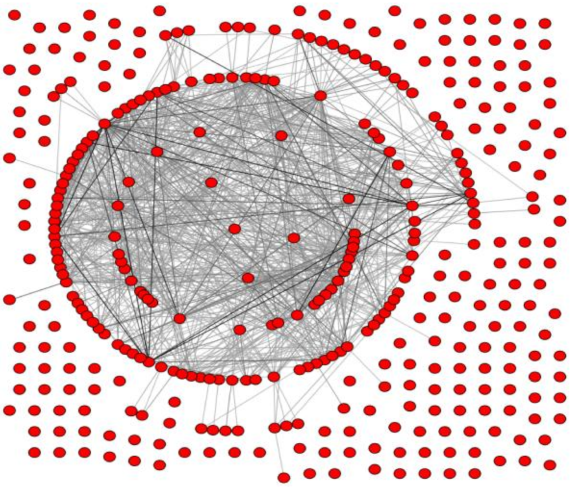

transporter activity 828 / 363

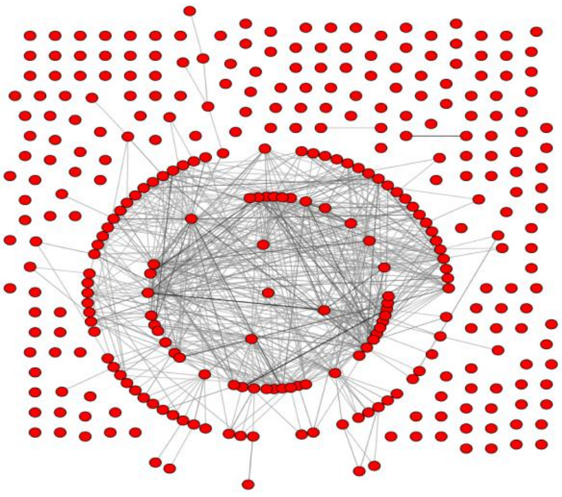

DNA binding 510 / 334

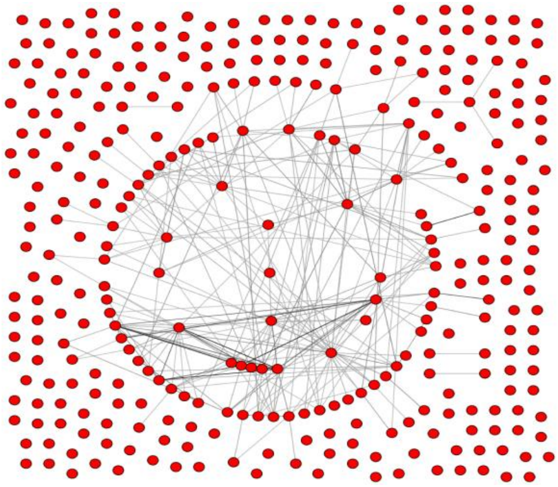

ribosome 236 / 333

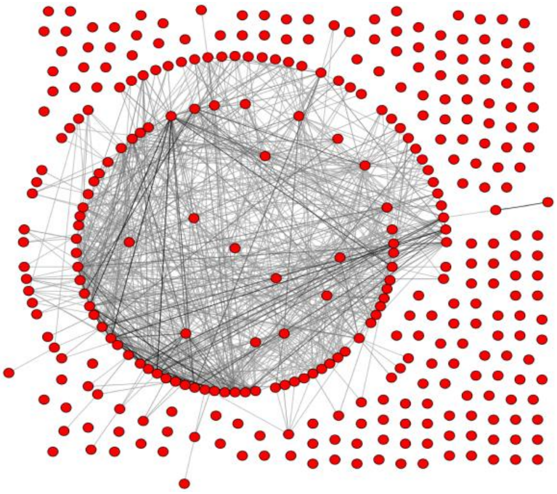

chromosome 673 / 329

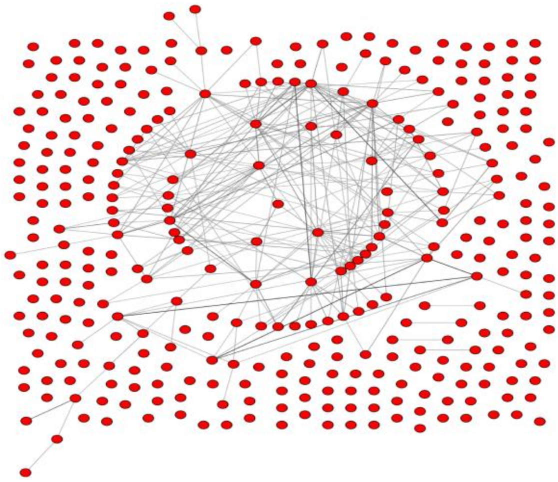

structural molecule activity 249 / 331

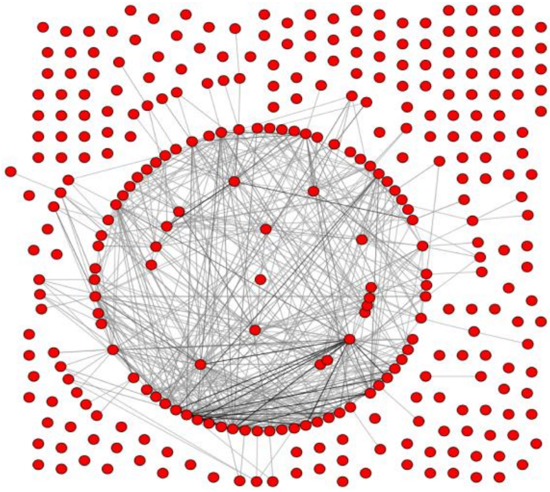

endomembrane system 461 / 318

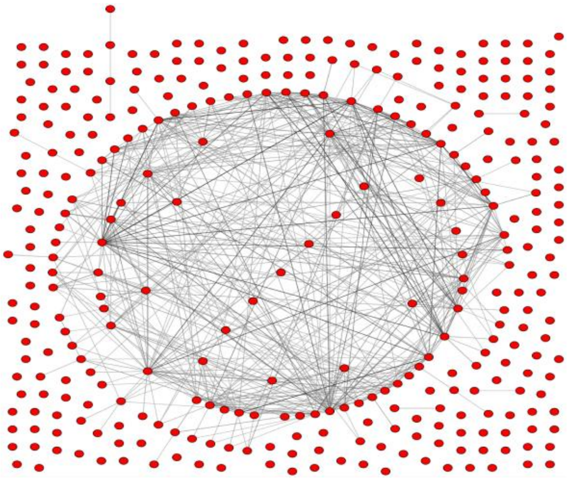

ribosome biogenesis 471 / 330

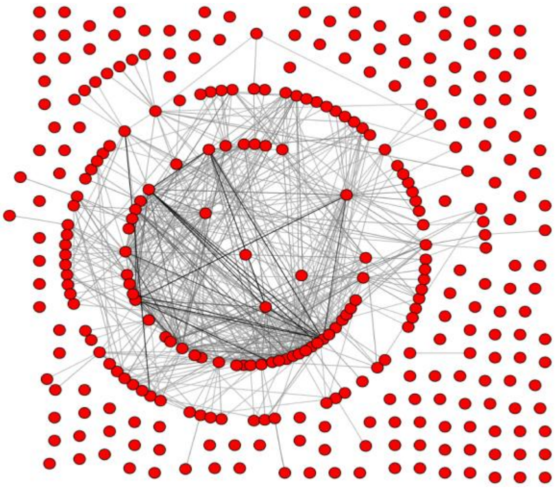

transcription regulator activity 472 / 312

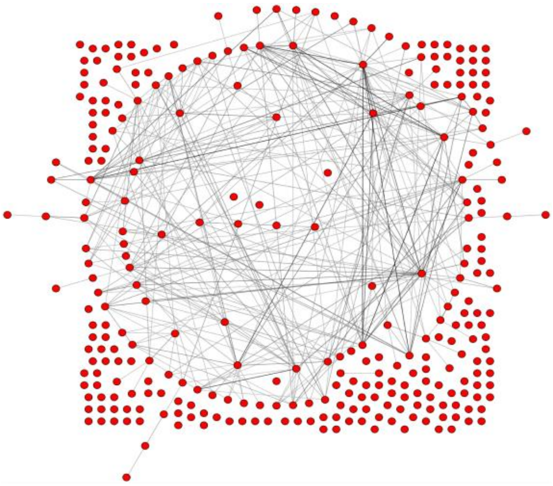

mitochondrial envelope 311 / 302

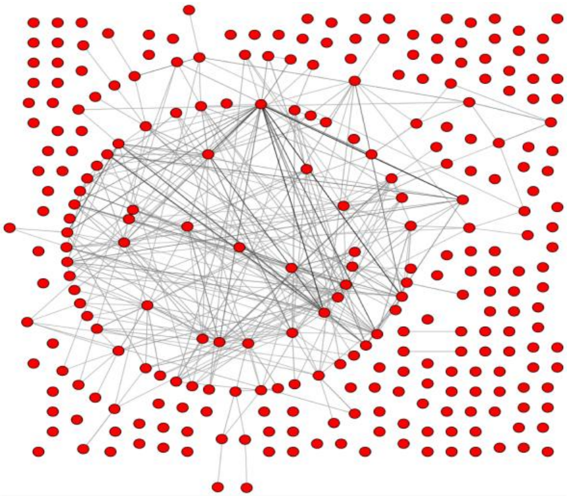

cellular membrane organization 298 / 271

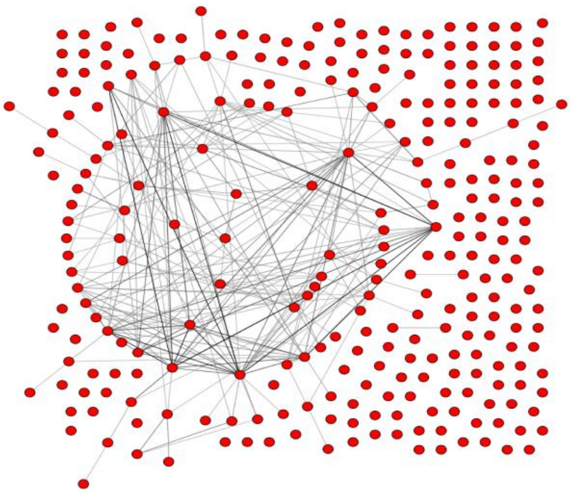

mitochondrion organization 229 / 283

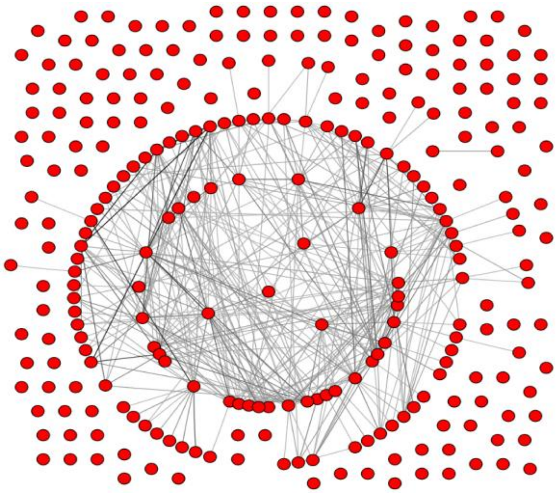

cellular carbohydrate metabolic process 350 / 265

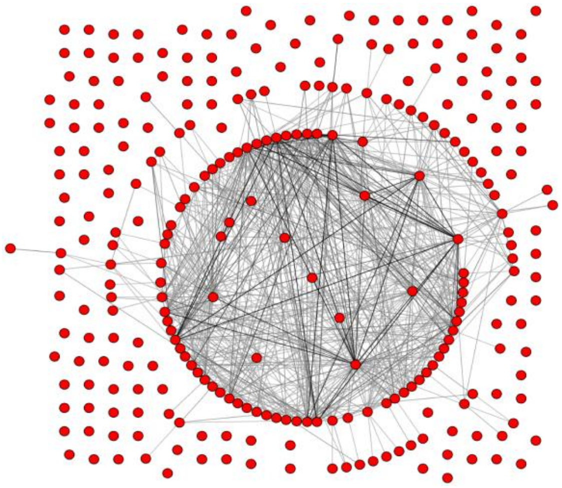

plasma membrane 579 / 279

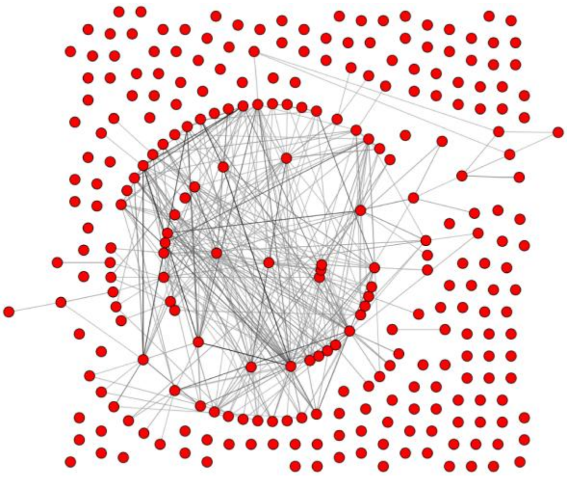

oxidoreductase activity 303 / 265

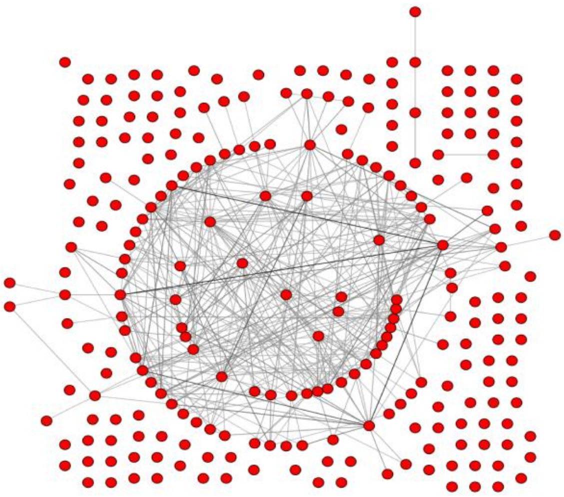

cellular amino acid and derivative metabolic process 331 / 250

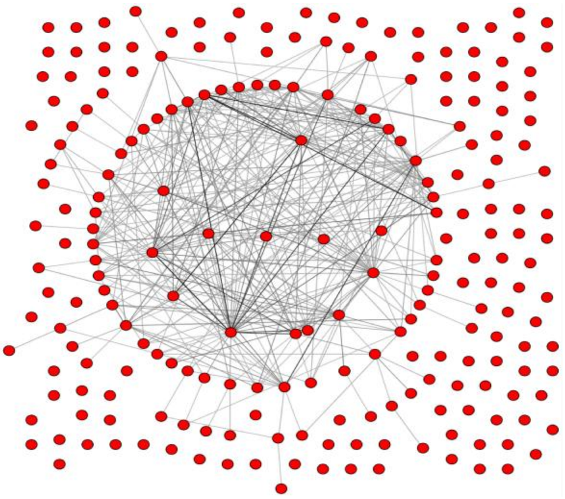

signal transduction 314 / 220

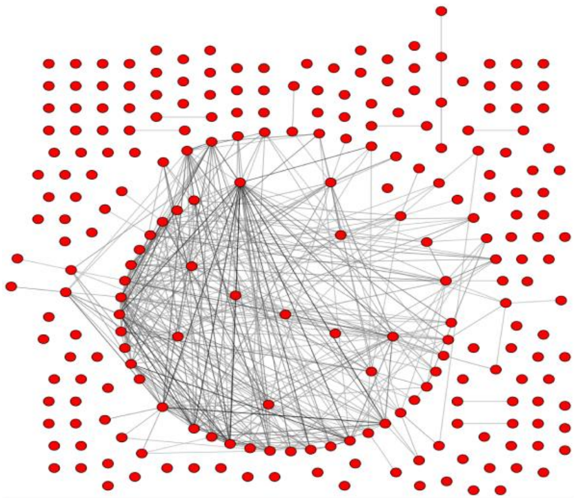

nucleolus 352 / 233

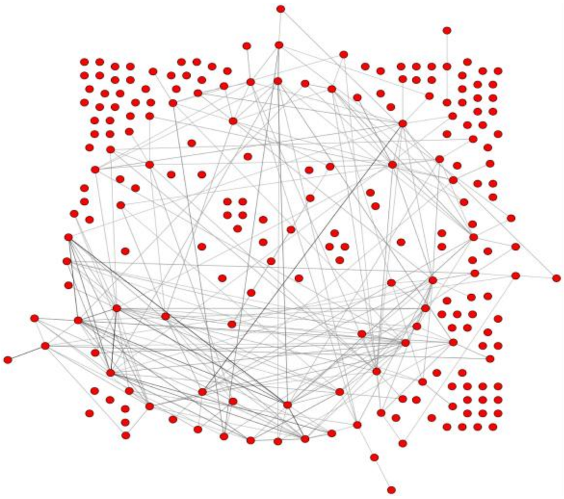

cytoskeleton organization 214 / 218

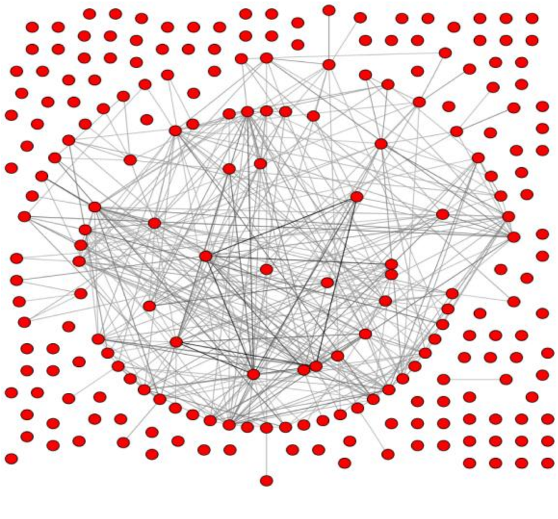

site of polarized growth 328 / 221

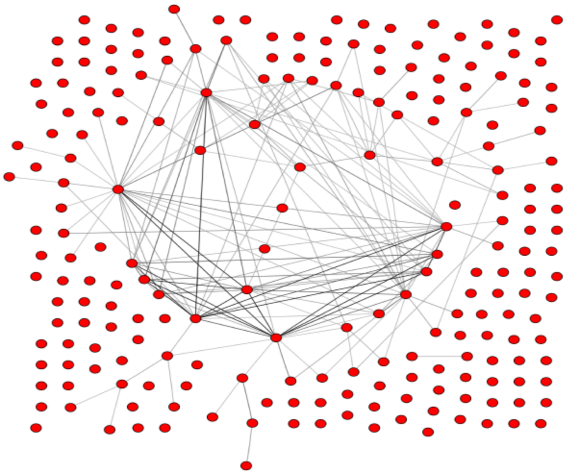

protein complex biogenesis 144 / 215

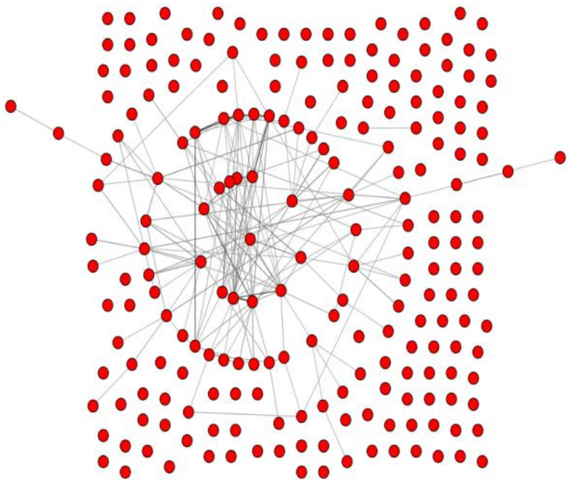

RNA binding 161 / 213

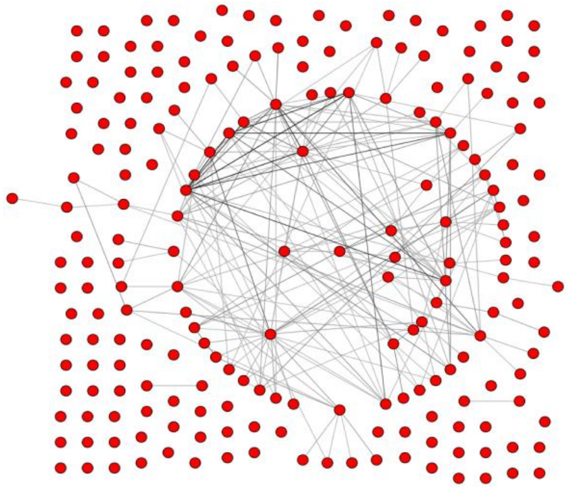

vacuole 198 / 204

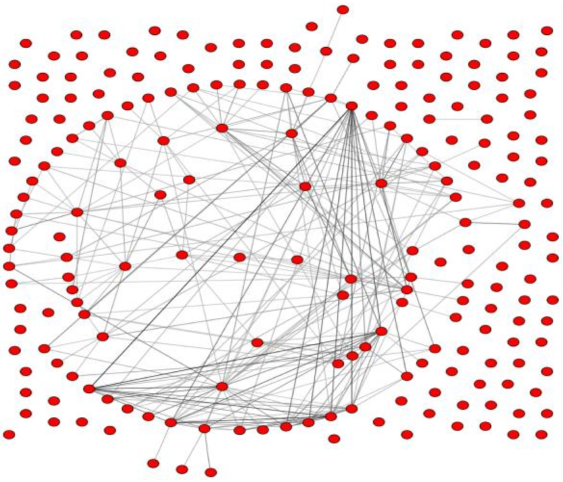

cellular lipid metabolic process 223 / 210

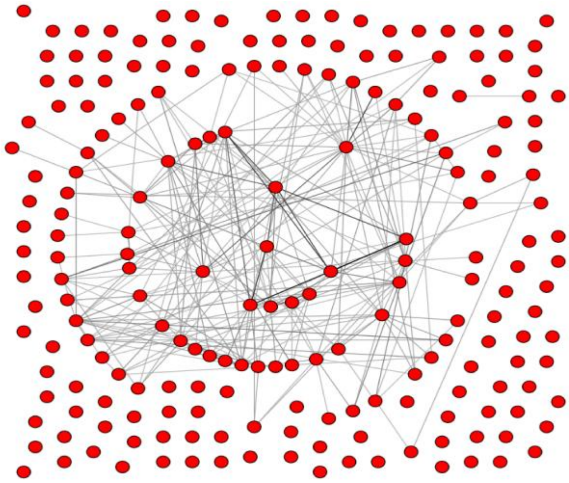

cytoskeleton 222 / 203

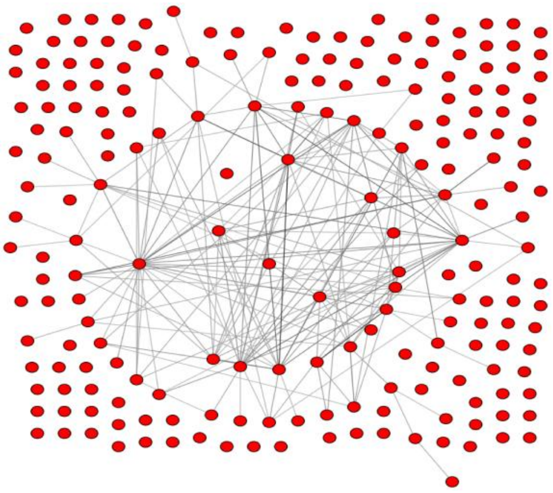

enzyme regulator activity 168 / 206

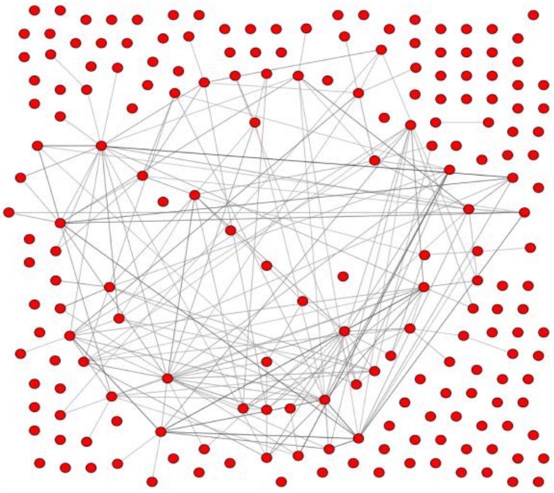

membrane fraction 165 / 195

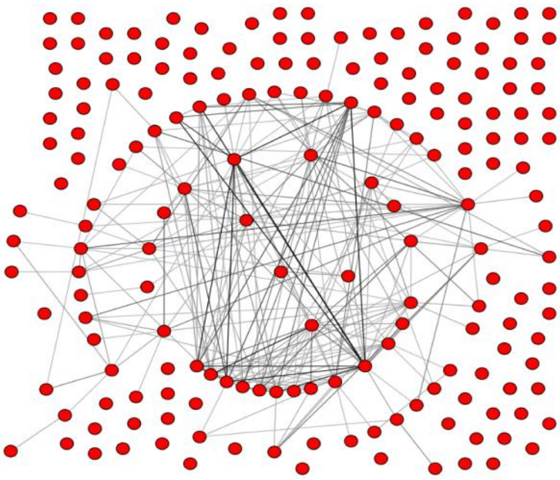

cellular protein catabolic process 216 / 184

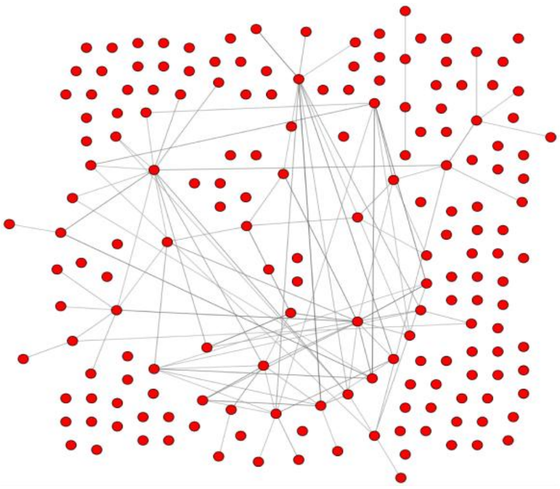

generation of precursor metabolites and energy 102 / 176

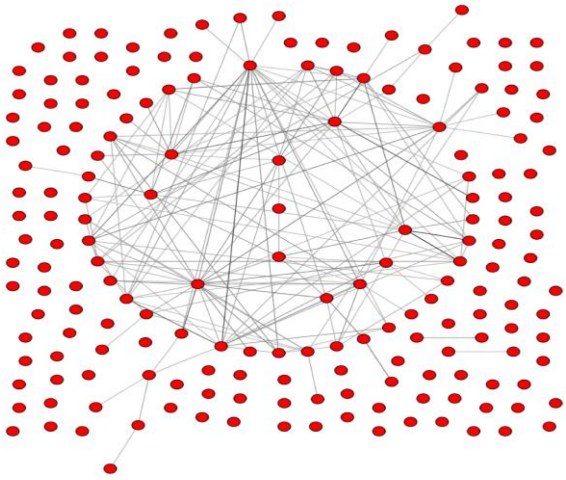

heterocycle metabolic process 164 / 183

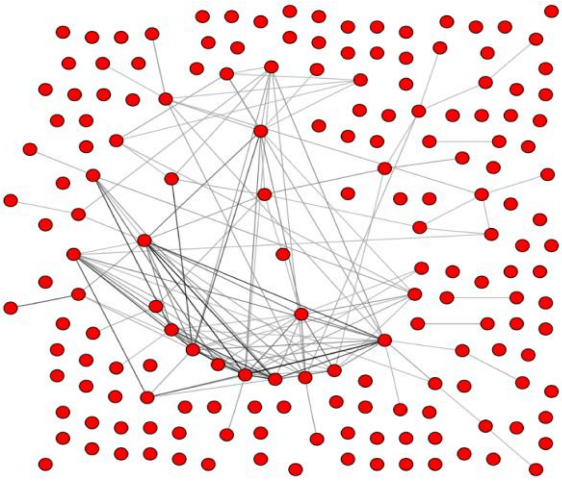

Golgi apparatus 128 / 171

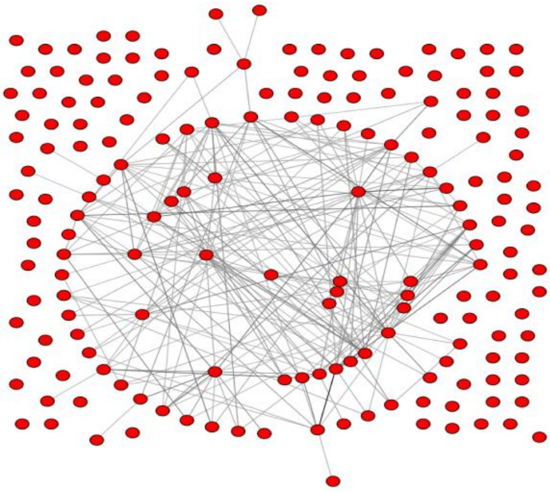

cellular bud 203 / 177

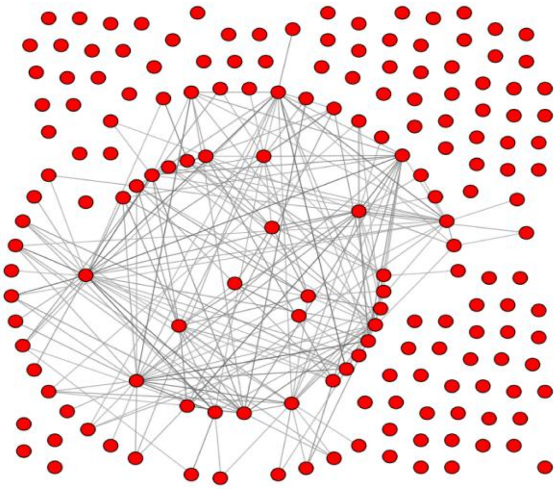

ligase activity 197 / 170

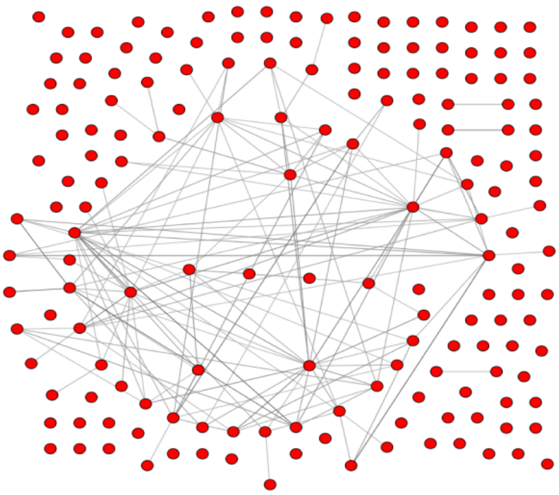

cofactor metabolic process 136 / 167

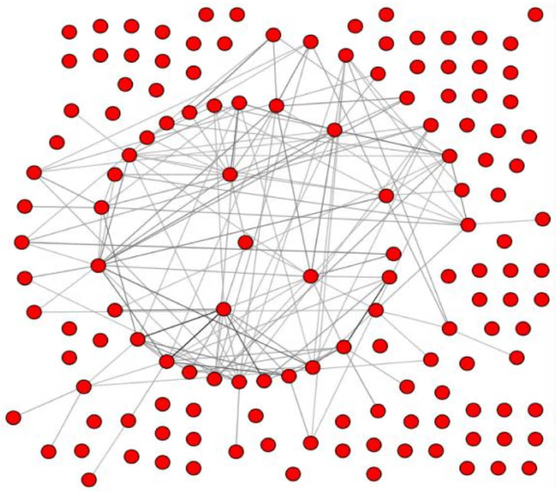

chromosome segregation 143 / 140

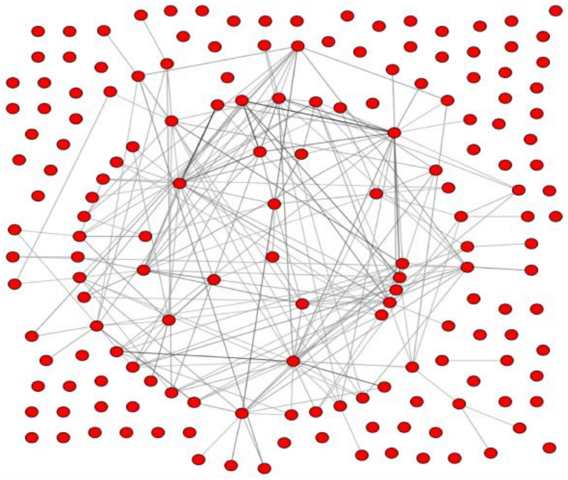

cellular component morphogenesis 199 / 167

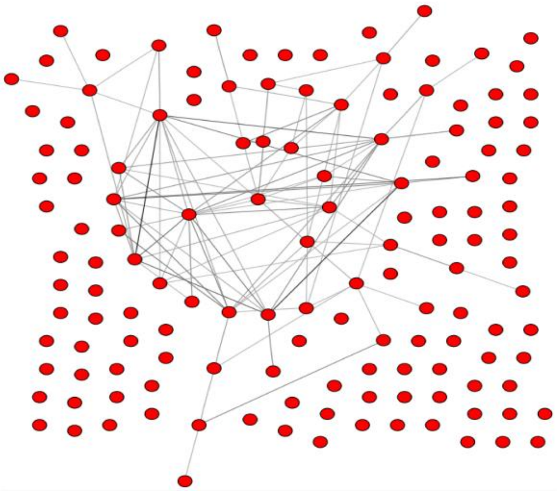

cellular homeostasis 97 / 138

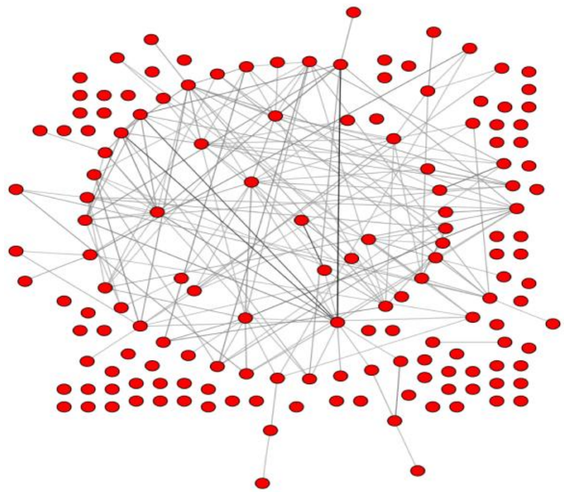

meiosis 170 / 152

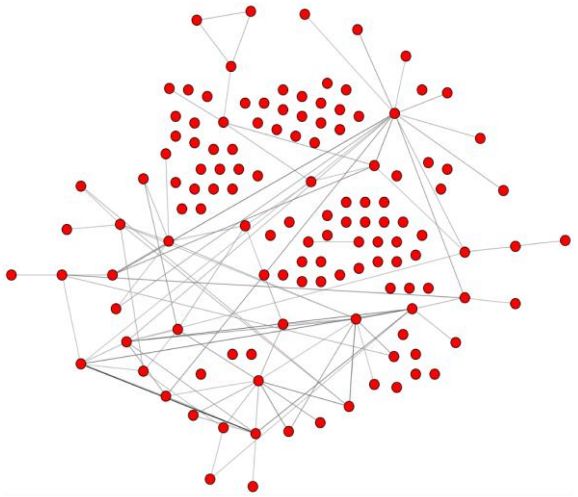

sporulation resulting in formation of a cellular spore 87 / 128

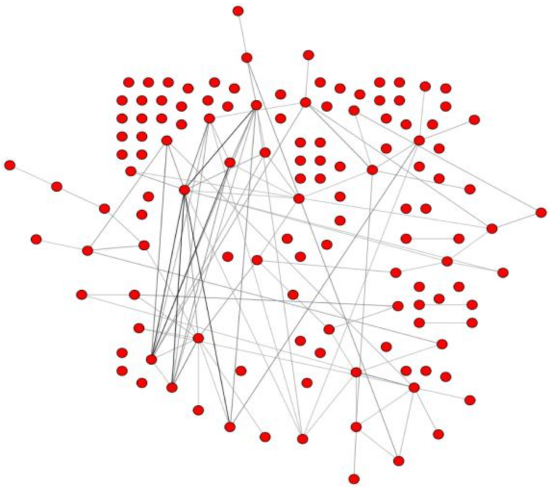

fungal-type cell wall organization 93 / 127

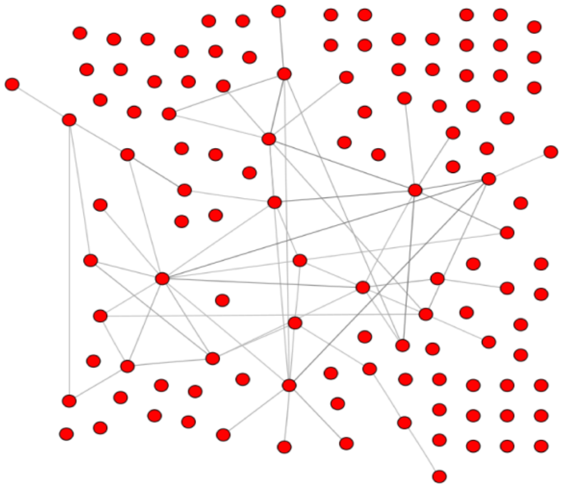

conjugation 57 / 117

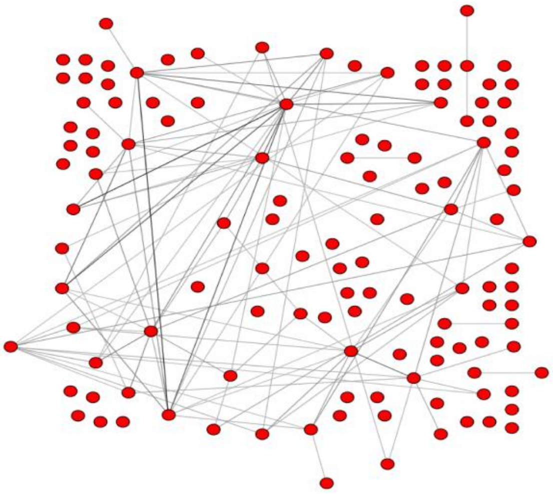

protein kinase activity 90 / 124

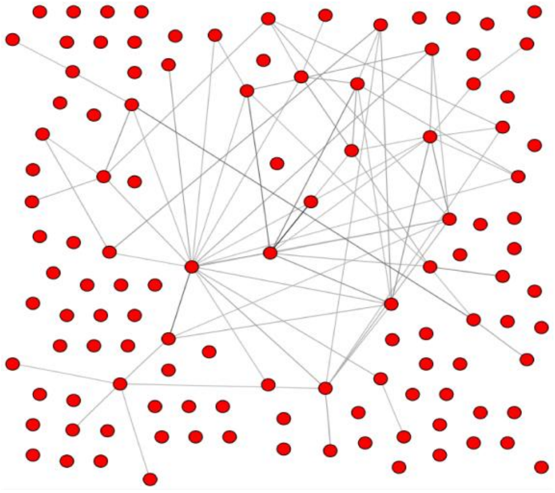

cytokinesis 74 / 116

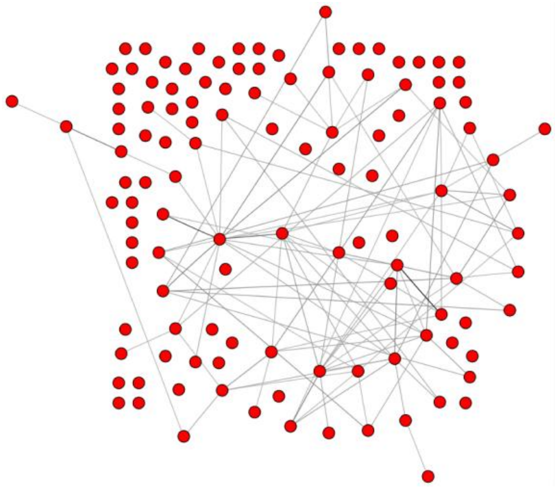

cell cortex 99 / 117

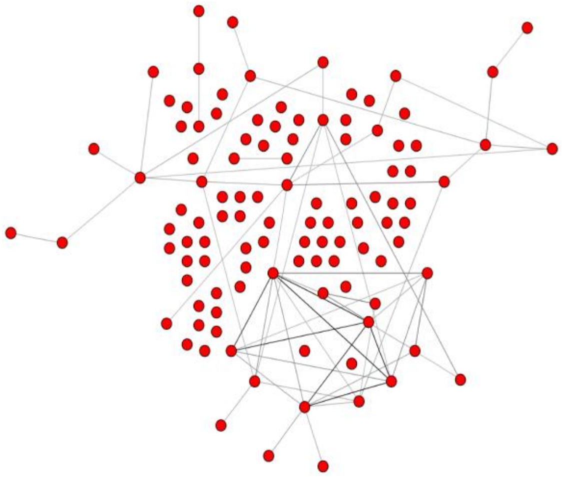

peptidase activity 60 / 109

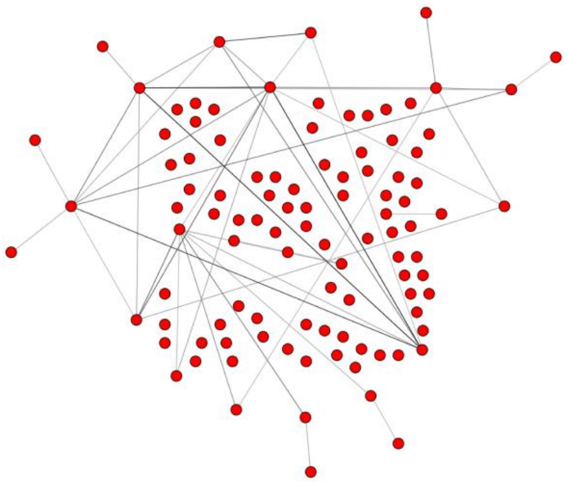

cytoplasmic membrane-bounded vesicle 46 / 102

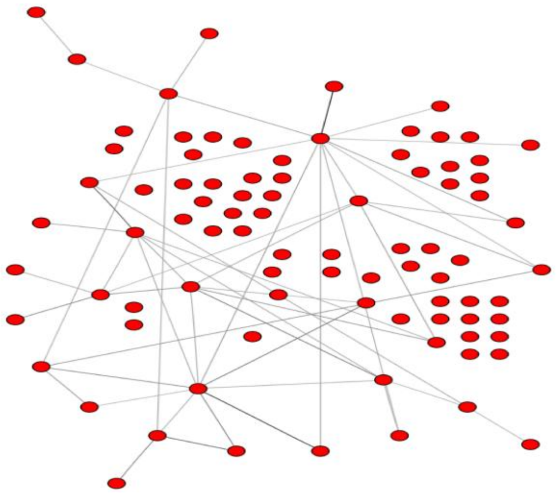

cell budding 47 / 86

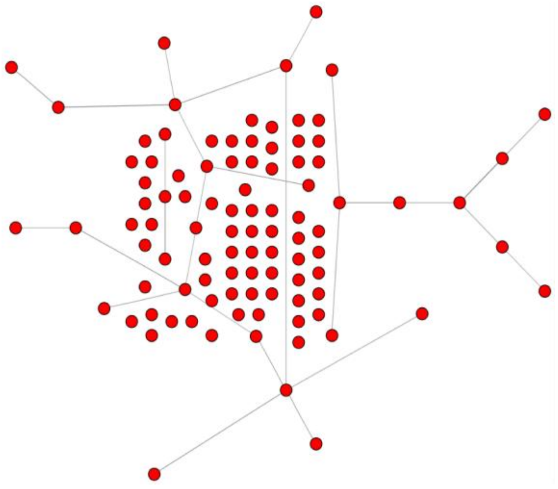

cell wall 28 / 96

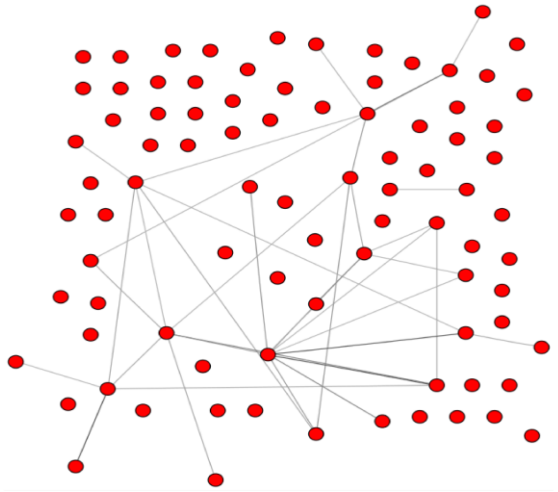

protein folding 37 / 86

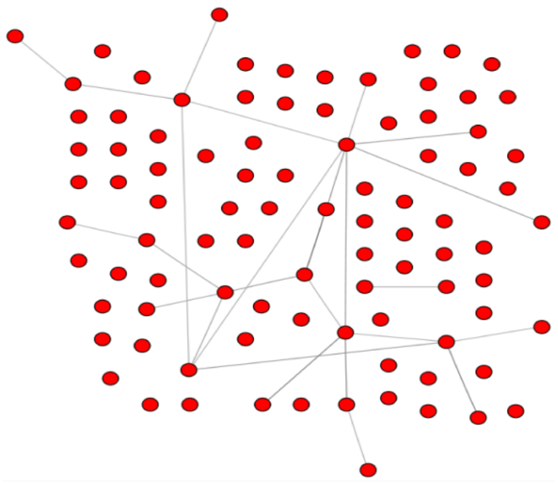

cellular respiration 26 / 92

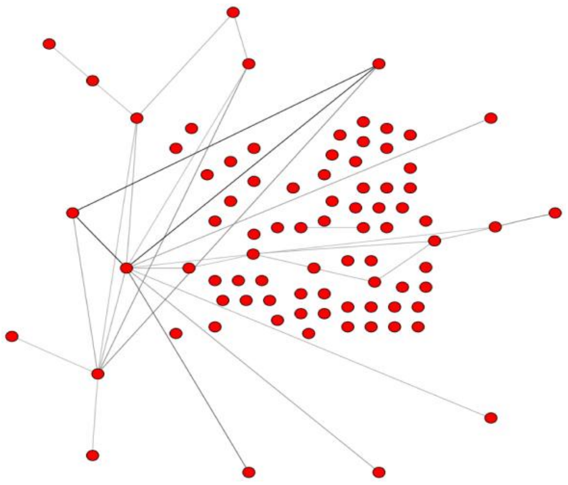

lyase activity 27 / 82

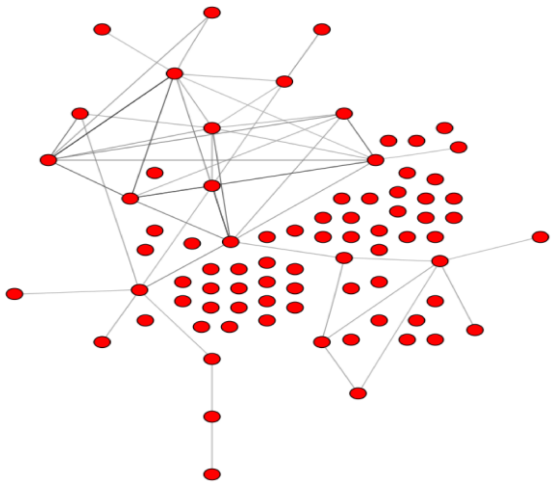

helicase activity 46 / 79

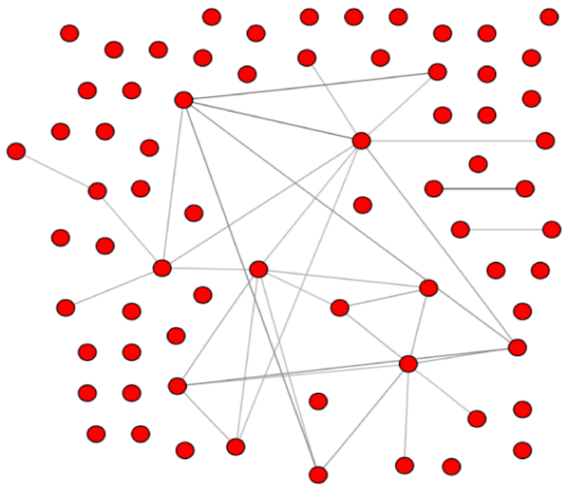

vesicle organization 28 / 70

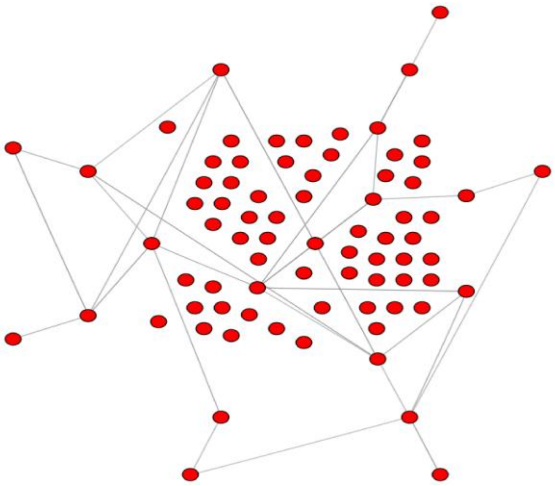

cellular aromatic compound metabolic process 28 / 76

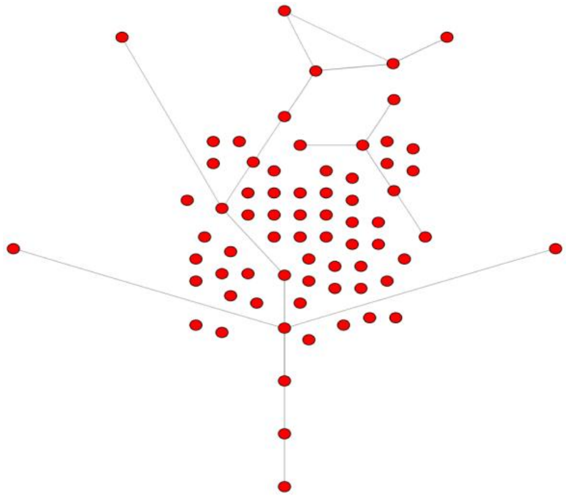

microtubule organizing center 16 / 70

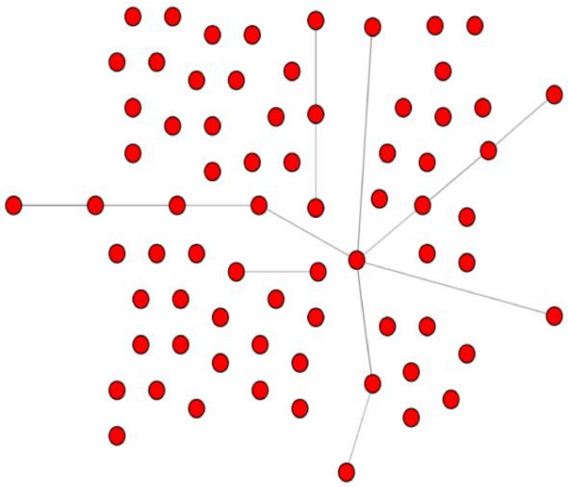

lipid binding 14 / 71

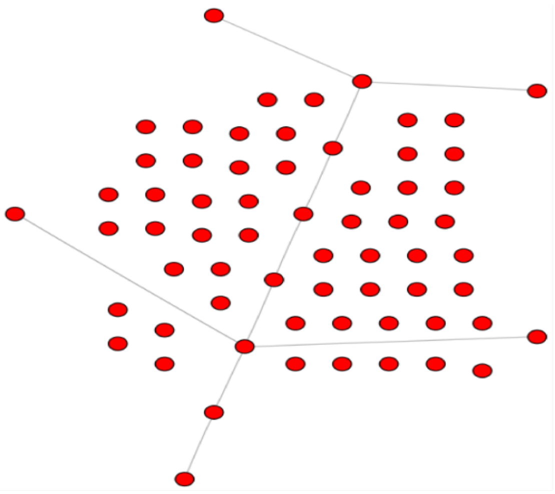

nucleotidyltransferase activity 10 / 64

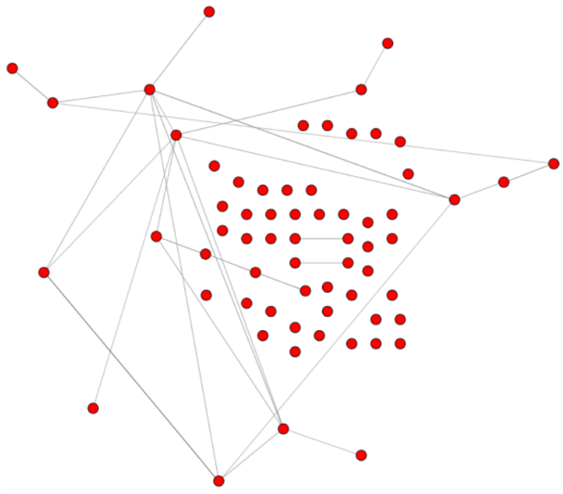

nucleus organization 29 / 64

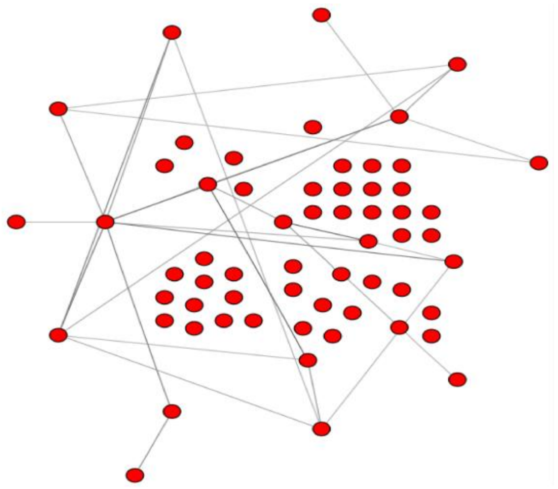

vitamin metabolic process 29 / 60

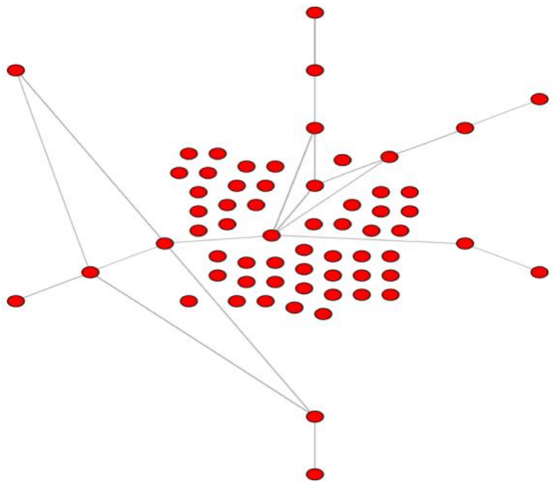

pseudohyphal growth 17 / 63

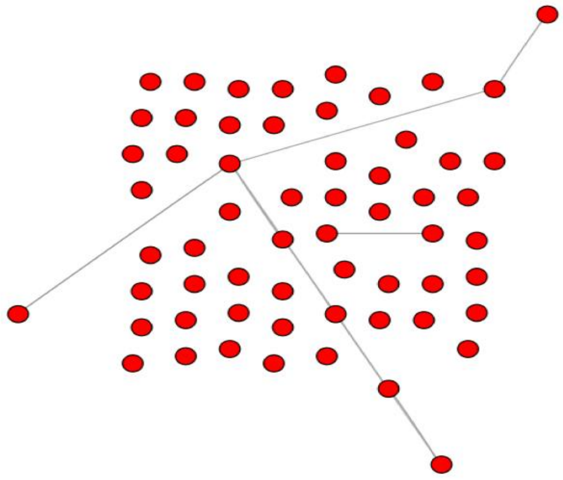

peroxisome 10 / 60

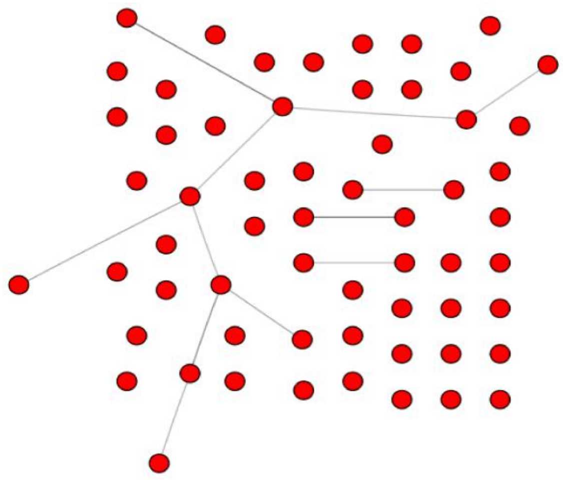

vacuole organization 12 / 60

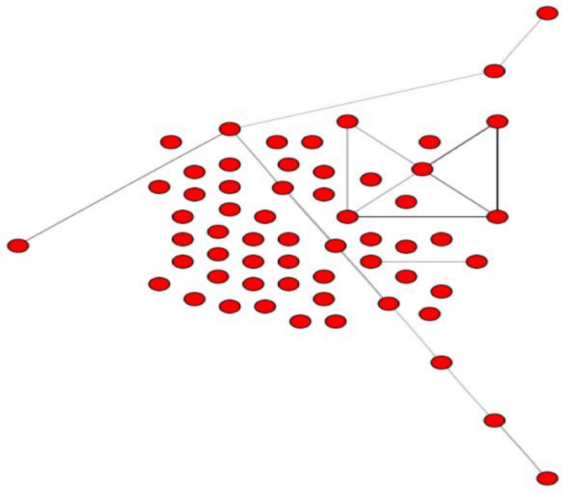

peroxisome organization 18 / 59

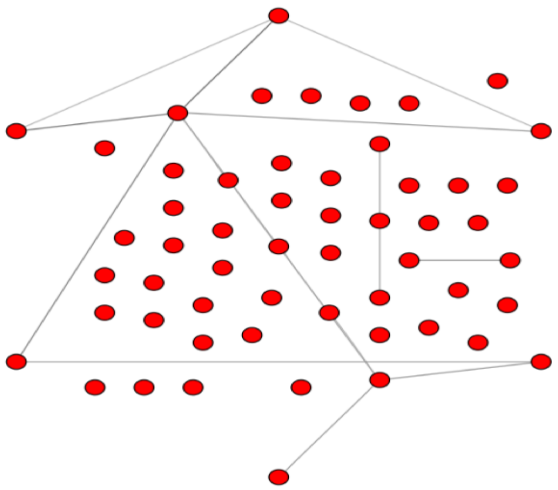

isomerase activity 17 / 55

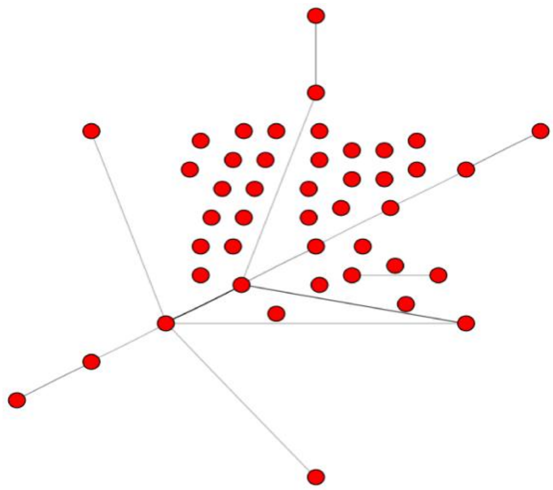

signal transducer activity 14 / 44

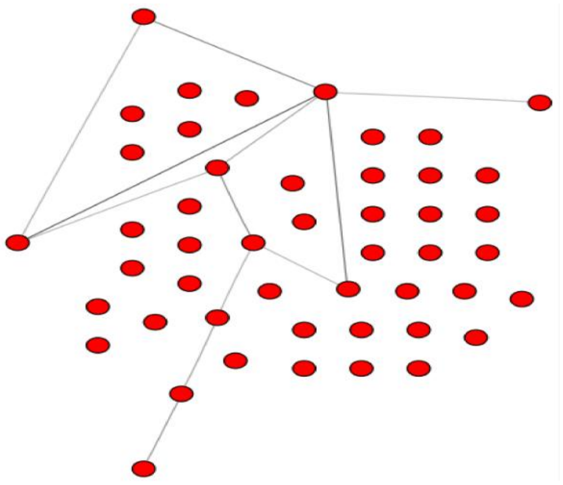

phosphoprotein phosphatase activity 12 / 48

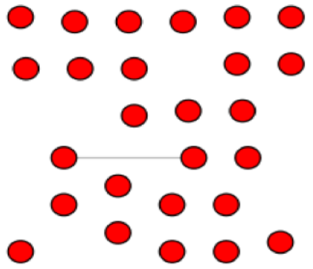

extracellular region 1 / 26

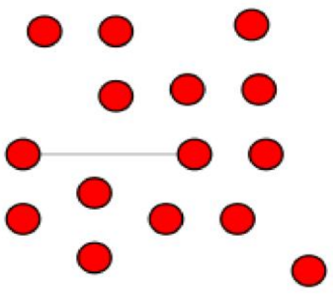

motor activity 1 / 15

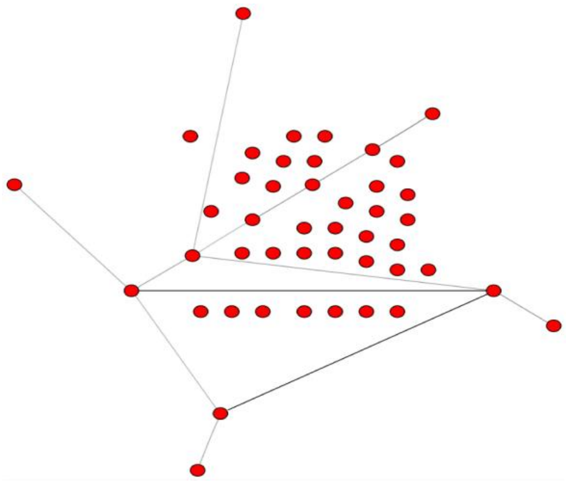

transposition 13 / 45

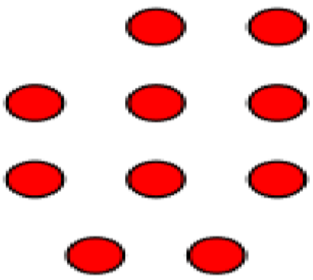

translation regulator activity 0 / 10
